# The Role of VP1 Amino Acid Residue 145 of Enterovirus 71 in Viral Fitness and Pathogenesis in a Cynomolgus Monkey Model

**DOI:** 10.1371/journal.ppat.1005033

**Published:** 2015-07-16

**Authors:** Chikako Kataoka, Tadaki Suzuki, Osamu Kotani, Naoko Iwata-Yoshikawa, Noriyo Nagata, Yasushi Ami, Takaji Wakita, Yorihiro Nishimura, Hiroyuki Shimizu

**Affiliations:** 1 Department of Virology II, National Institute of Infectious Diseases, Tokyo, Japan; 2 Department of Pathology, National Institute of Infectious Diseases, Tokyo, Japan; 3 Division of Experimental Animal Research, National Institute of Infectious Diseases, Tokyo, Japan; 4 Division of Infectious Diseases, The Children's Hospital of Philadelphia, Philadelphia, Pennsylvania, United States of America; University of Pittsburgh, UNITED STATES

## Abstract

Enterovirus 71 (EV71), a major causative agent of hand, foot, and mouth disease, occasionally causes severe neurological symptoms. We identified P-selectin glycoprotein ligand-1 (PSGL-1) as an EV71 receptor and found that an amino acid residue 145 in the capsid protein VP1 (VP1-145) defined PSGL-1-binding (PB) and PSGL-1-nonbinding (non-PB) phenotypes of EV71. However, the role of PSGL-1-dependent EV71 replication in neuropathogenesis remains poorly understood. In this study, we investigated viral replication, genetic stability, and the pathogenicity of PB and non-PB strains of EV71 in a cynomolgus monkey model. Monkeys were intravenously inoculated with cDNA-derived PB and non-PB strains of EV71, EV71-02363-EG and EV71-02363-KE strains, respectively, with two amino acid differences at VP1-98 and VP1-145. Mild neurological symptoms, transient lymphocytopenia, and inflammatory cytokine responses, were found predominantly in the 02363-KE-inoculated monkeys. During the early stage of infection, viruses were frequently detected in clinical samples from 02363-KE-inoculated monkeys but rarely in samples from 02363-EG-inoculated monkeys. Histopathological analysis of central nervous system (CNS) tissues at 10 days postinfection revealed that 02363-KE induced neuropathogenesis more efficiently than that induced by 02363-EG. After inoculation with 02363-EG, almost all EV71 variants detected in clinical samples, CNS, and non-CNS tissues, possessed a G to E amino acid substitution at VP1-145, suggesting a strong *in vivo* selection of VP1-145E variants and CNS spread presumably in a PSGL-1-independent manner. EV71 variants with VP1-145G were identified only in peripheral blood mononuclear cells in two out of four 02363-EG-inoculated monkeys. Thus, VP1-145E variants are mainly responsible for the development of viremia and neuropathogenesis in a non-human primate model, further suggesting the *in vivo* involvement of amino acid polymorphism at VP1-145 in cell-specific viral replication, *in vivo* fitness, and pathogenesis in EV71-infected individuals.

## Author Summary

Recently, large outbreaks of hand, foot, and mouth disease, including fatal neurological cases in young children primarily because of enterovirus 71 (EV71) have been reported, particularly in the Asia Pacific regions where the disease poses a serious threat to public health. Based on mutational and structural analyses of EV71, we identified amino acid residue 145 of the capsid protein VP1 (VP1-145) as a critical molecular determinant for the binding of EV71 to a specific cellular receptor, human P-selectin glycoprotein ligand-1 (PSGL-1). VP1-145 is highly variable among EV71 isolates and has been identified as a potential neurovirulence determinant in humans and experimental mouse models. To elucidate the *in vivo* involvement of PSGL-1-depentent replication and pathogenesis, we investigated viral replication, genetic stability, and the pathogenicity of the PSGL-1-binding (PB) and PSGL-1-nonbinding (non-PB) strains of EV71 in a cynomolgus monkey model. After the intravenous inoculation with the PB strain, viruses found to be highly mutated at VP1-145 with resultant VP1-145E variants (non-PB) inducing viremia and neuropathogenesis, presumably in a PSGL-1-independent manner. VP1-145G variants were identified only in peripheral blood mononuclear cells from two PB-inoculated monkeys. Our study provides new insights into the interplay between virus, receptors, and host in EV71-infected individuals.

## Introduction

Enterovirus 71 (EV71) is a non-enveloped positive-stranded RNA virus belonging to the species *Enterovirus A* of the genus *Enterovirus* in the family *Picornaviridae*. The EV71 RNA genome is enclosed within an icosahedral capsid comprising 60 structural protein subunits (protomers), each containing four viral structural proteins, VP1-VP4 [1, 2]. According to the molecular epidemiological analysis of the capsid VP1 sequence, EV71 was previously classified into the three genogroups A, B (subgenogroups B1–B5), and C (subgenogroups C1–C5) [3, 4]. Recently, several additional genogroups of EV71 were identified as genogroups D, E, and F [5].

EV71 is a major causative agent of hand, foot, and mouth disease (HFMD) along with coxsackievirus A16 (CVA16) and coxsackievirus A6 (CVA6) [6, 7]. EV71 usually causes mild or subclinical infection. However, in some patients, EV71 may cause severe neurological symptoms, including meningitis, brainstem encephalitis, poliomyelitis-like paralysis, pulmonary edema, and death. As recent EV71 outbreaks in the Asia-Pacific region, including Malaysia, Taiwan, China, Cambodia, and Vietnam, have involved millions of children (almost all under 5 years old) including thousands of fatal cases, EV71 poses a threat to global public health [4, 8–10]. Recently, a growing number of viral and host factors associated with EV71 infection have been reported [11–14]; however, no conclusive risk factors for neuropathogenesis have yet been identified. In the previous studies, two molecules, scavenger receptor class B, member 2 (SCARB2) [15] and P-selectin glycoprotein ligand-1 (PSGL-1) [16] were identified as functional receptors for EV71. Subsequently, other cell surface molecules, including heparan sulfate [17], annexin II [18], sialic acid [19], dendritic cell-specific ICAM3-grabbing non-integrin (DC-SIGN) [20], and vimentin [21] have been identified as EV71-binding molecules involved in the early stages of EV71 infection. SCARB2 is expressed on the membrane of various cells and tissues, and involved in the endocytic transport mechanism from the ER to lysosomes [22, 23]. Expression of human SCARB2 allows non-susceptible mouse L929 cells (L-SCARB2) to support EV71 replication and development of cytopathic effects [15]. All EV71 strains examined and coxsackievirus A7, A14, and A16 replicate in L-SCARB2 and SCARB2-positive RD cells in a SCARB2-dependent manner [15, 24]. Furthermore, transgenic mice expressing human SCARB2 (SCARB2-Tg) are more susceptible to EV71 and developed neurological disorders following EV71 infection [25, 26].

We have identified another functional cellular receptor, PSGL-1 [16], expressed on lymphocytes that plays a critical role in tethering and rolling during recruitment of leukocytes from blood vessels into inflamed tissues [27, 28]. In contrast to SCARB2-Tg mice, human PSGL-1 expressing transgenic mice are not susceptible to EV71 infection [29]. Independent of genogroup/subgenogroup, some EV71 strains have been found to bind PSGL-1 and infect Jurkat T cells in a PSGL-1-dependent manner whilst other strains do not. Thus, EV71 strains are classified into two distinct phenotypes according to PSGL-1-binding capability; PSGL-1-binding (PB) and PSGL-1-nonbinding (non-PB) strains [16]. Through molecular epidemiological, structural, and mutational analyses of EV71, we recently demonstrated an amino acid residue 145 of the capsid protein VP1 (VP1-145) as a critical molecular determinant of the binding of PB EV71 strains to PSGL-1 [30]. VP1-145 is located within the DE loop at the center of the 5-fold mesa of EV71 virions and functions as a molecular switch to change the binding to PSGL-1 by regulating the orientation of the side chain of lysine at VP1-244 [30]. Accordingly, EV71 strains with either G or Q at VP1-145 (VP1-145G or VP1-145Q) exhibit a PB phenotype and those with E at VP1-145 (VP1-145E) have a non-PB phenotype regardless of EV71 genogroup. VP1-145 is a variable amino acid residue among the field EV71 isolates and has been identified as a major site of positive selection in the molecular evolution of EV71 [31–33]. Based on the available VP1 sequences of EV71 in the GenBank database, VP1-145E isolates were most predominant (81%), and VP1-145G (9%) and VP1-145Q (9%) isolates were less common than VP1-145E [30].

Furthermore, substitution of VP1-145G or VP1-145Q to VP1-145E was found to be responsible for murine adaptation and/or virulence, either alone or in combination with other amino acids, demonstrating VP1-145E strains of EV71 are more virulent than either VP1-145G or VP1-145Q (VP1-145G/Q) strains in different mouse models [34–37]. On the other hand, recent molecular epidemiological studies have suggested that VP1-145G/Q isolates are more frequently detected in cases with severe neurological disease in humans than VP1-145E isolates [38–41]. These apparently contradictory findings in humans and mouse models are yet to be resolved.

To elucidate the *in vivo* involvement of PSGL-1-dependent replication and pathogenesis, and the role of amino acid polymorphism at VP1-145, we investigated viral replication, pathogenicity, and genetic stability of PB (VP1-145G) and non-PB (VP1-145E) strains of EV71 in a cynomolgus monkey model, a more reliable animal model than mouse models due to greater homology between primate and human PSGL-1 molecules than mouse. We found that, following inoculation of monkeys with the PB strain of EV71, the PB strain with VP1-145G frequently underwent mutation at VP1-145 from G to E (VP1-145E), and the resultant VP1-145E variants were capable of inducing viremia and neuropathology, presumably in a PSGL-1-independent manner. Conversely, PB variants with VP1-145G were identified only in peripheral blood mononuclear cells (PBMCs) in two out of four PB-inoculated monkeys in the later stages of infection, suggesting potential involvement of PSGL-1-dependent EV71 replication of PB variants in cell-specific viral replication and pathogenesis in EV71-infected individuals.

## Results

### Preparation of PB and non-PB strains of EV71

**Fig 1:**
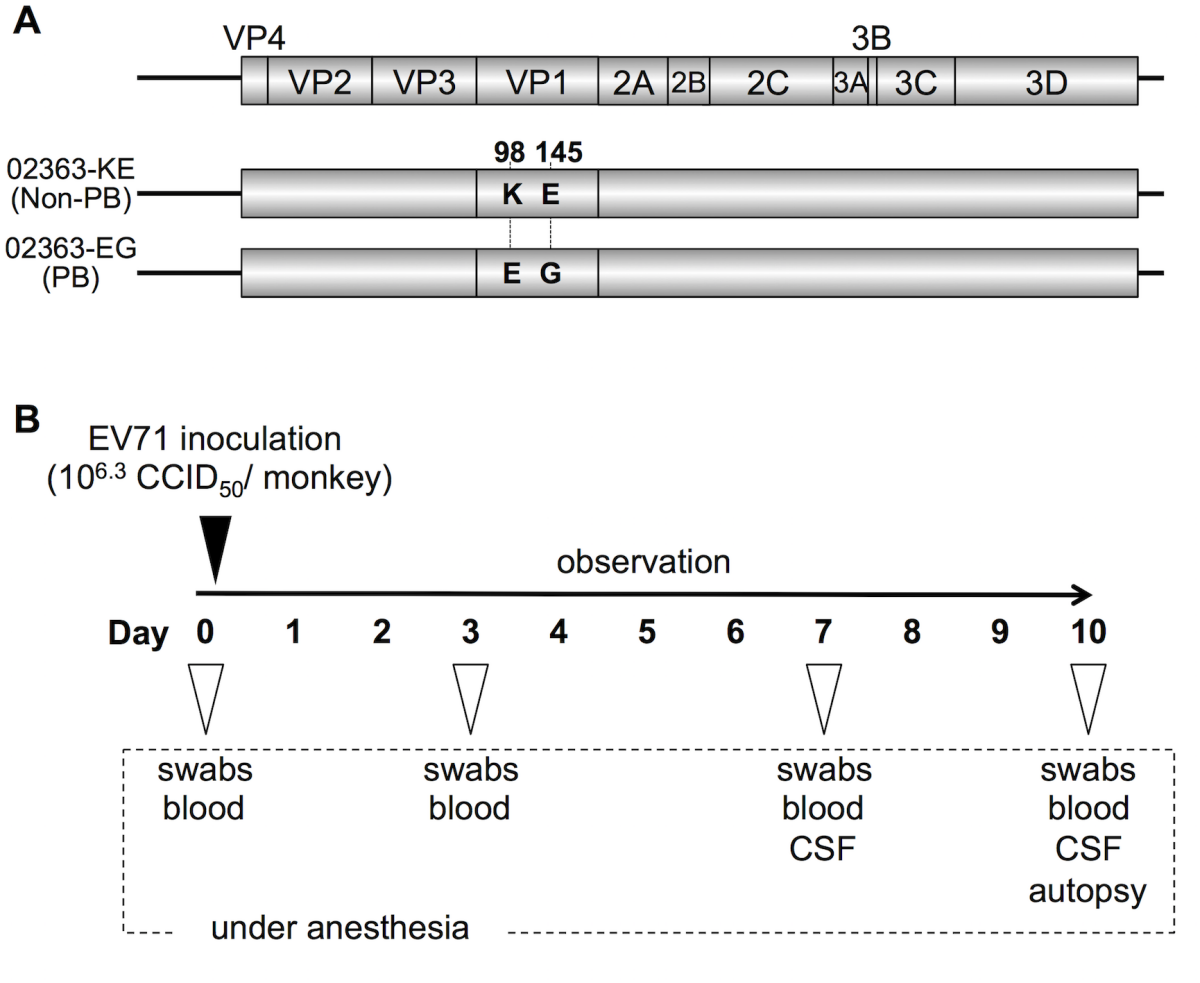
Experimental infection of cynomolgus monkeys with EV71 strains. (A) Scheme of cDNA-derived PSGL-1-binding (PB) and nonbinding (non-PB) strains of EV71 with two amino acid substitutions at VP1-98 and VP1-145. (B) Experimental schedule. Four monkeys were intravenously inoculated with 1 ml containing 10^6.3^ CCID_50_ of EV71-02363-KE (non-PB) or EV71-02363-EG (PB) strain at Day 0 and the clinical manifestations were observed daily for 10 days. Under anesthesia, clinical samples were collected on indicated days and postmortem tissue samples were collected at 10 days postinfection.

To prepare cDNA-derived PB and non-PB strains of EV71, we used an infectious molecular clone of the 02363 strain of EV71 (subgenogroup C1; GenBank accession No. AB747375). The cDNA-derived 02363 strain contains a combination of VP1-98**K** and VP1-145**E** (EV71-02363-**KE** strain). VP1-145E has been identified as a single determinant of the non-PB phenotype by using a series of 02363-derived EV71 mutants in which amino acid substitutions were introduced at VP1-145 and/or VP1-98 [30]. VP1-98 (98E or 98K) was not directly responsible for the PB phenotype. In the previous studies, VP1-145E of EV71 mutants was found to be unstable and rapidly reverted to VP1-145G during virus passage in RD cells [30, 35]. However, no apparent reversions at VP1-145 were observed in RD cells for the 02363-KE strain or the 02363-derived strain with VP1-98**E** and VP1-145**G** mutations (EV71-02363-**EG** strain). Therefore, we choose the cDNA-derived EV71-02363-KE and EV71-02363-EG strains, with two amino acid differences at VP1-98 and VP1-145, as representative non-PB and PB strains, respectively (Fig 1A) for use in a cynomolgus monkey model.

To minimize the emergence of revertants and quasi-species during virus passages in cell culture, viral stocks were prepared from a single passage in RD cells following RNA transfection of RD cells. Furthermore, we confirmed 100% sequence identity of virus stocks against original cDNA clones by direct sequencing of RT-PCR products covering the full-capsid sequence of EV71 (GenBank accession No. AB747375). Both cDNA-derived PB and non-PB strains (02363-EG and 02363-KE, respectively) had similar growth kinetics in RD cells [30]. The 02363-EG strain was capable of replication in Jurkat T cells in a PSGL-1-dependent manner but 02363-KE did not replicate in T cells, confirming the PB and non-PB phenotypes [30].

### Clinical symptoms in cynomolgus monkeys inoculated with PB and non-PB strains

**Fig 2:**
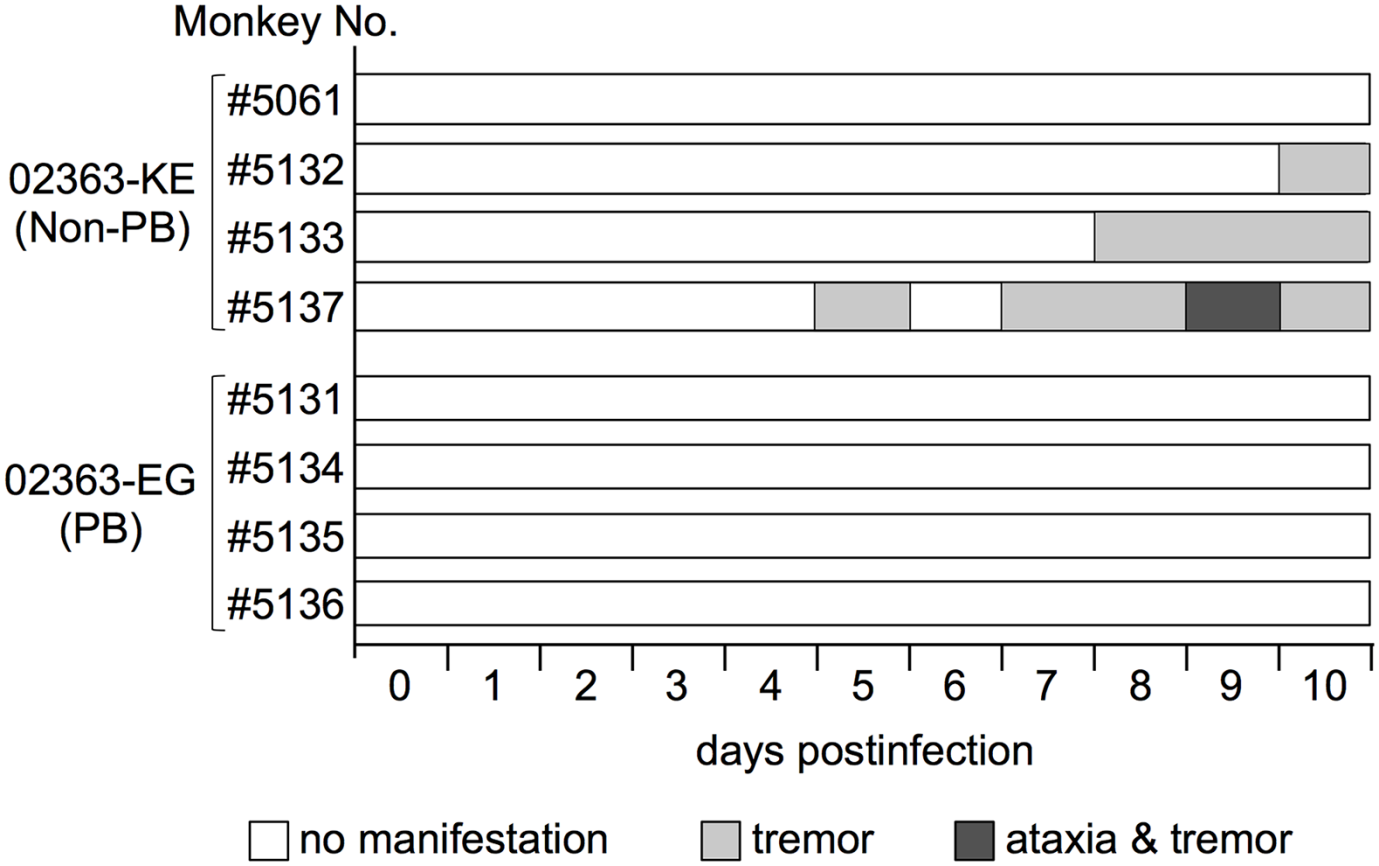
Time-course of clinical observations. Four monkeys (#5061, #5132, #5133, and #5137) were inoculated with the EV71-02363-KE (non-PB) strain and four monkeys (#5131, #5134, #5135, and #5136) were inoculated with the EV71-02363-EG (PB) strain. One monkey (#5061) exhibited decreased limb grip strength for 5–10 days postinfection (p.i.), without apparent paralysis, tremor, or ataxia.

We previously established a cynomolgus monkey model of acute viremia by using intravenous inoculation of EV71 to investigate the neuropathology of different EV71 strains in non-human primates [42–44]. Using this model in the present study, we compared the neuropathogenicity of EV71-02363-EG (PB) and EV71-02363-KE (non-PB) strains. As shown in Fig 1B, four monkeys were intravenously inoculated with 10^6.3^ CCID_50_ of 02363-EG or 02363-KE strain and clinical manifestations of the monkeys were observed daily for 10 days. During the observation period, no monkeys in either group demonstrated severe clinical manifestations. In the 02363-KE-inoculated group, three out of four monkeys (#5132, #5133, and #5137) exhibited tremor and/or ataxia in the later stages of infection (Fig 2). None of the 02363-EG-inoculated monkeys had apparent neurological manifestations. Neither exanthema (HFMD-like symptoms) nor pulmonary edema was observed in either group. Thus, mild neurological symptoms were found predominantly in the 02363-KE-inoculated monkeys but not in the EG-inoculated ones.

Serum samples were collected at 3, 7, and 10 days p.i., from inoculated monkeys to determine the serum neutralizing antibody titers against the inoculated EV71 strain. Neutralizing antibody titers were induced at 7–10 days p.i. in both groups, even in the four 02363-EG-inoculated monkeys that exhibited no apparent clinical symptoms with asymptomatic EV71 infection (S1 Fig).

### Kinetics of lymphocytes in peripheral blood

**Fig 3:**
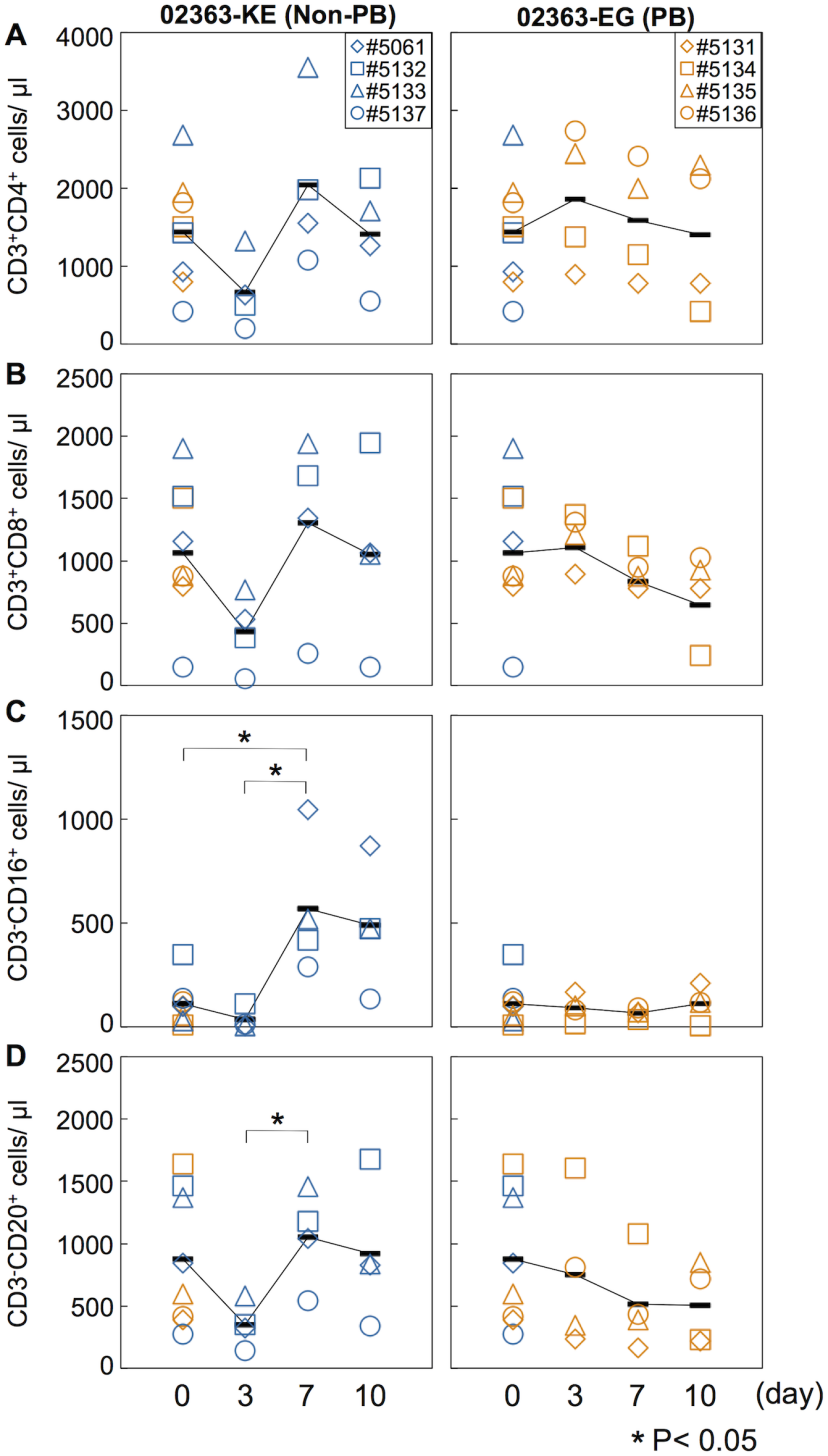
Kinetics of lymphocytes in peripheral blood. Number of (A) CD4^+^ T-lymphocytes, (B) CD8^+^ T-lymphocytes, (C) CD16^+^ lymphocytes (NK cells), and (D) CD20^+^ lymphocyte (B cells) in peripheral blood collected from monkeys inoculated with EV71-02363-KE (non-PB; left) or EV71-02363-EG (PB; right) were analyzed by flow cytometry. Pre-inoculation lymphocyte numbers in all eight monkeys are shown at Day 0 and the average was used as the uninfected control. Numbers of lymphocytes in peripheral blood, collected at 3, 7, and 10 days postinfection from the 02363-KE-iniculated group are indicated in blue and those from the 02363-EG-inoculated group are indicated in orange. Averages of lymphocyte numbers are indicated as black solid line plot. Significant differences between the preinfection control (eight monkeys at Day 0) and postinfection (four monkeys) at indicated days were determined using Student's *t*-test and *P* values < 0.05 are marked by asterisks Significant differences in lymphocyte numbers between 02363-KE- and 02363-EG-inoculated groups at indicated days are shown in S2 Fig.

In a cell culture system, the 02363-EG strain is capable of replication in Jurkat T cells in a PSGL-1-dependent manner whilst the 02363-KE strain is not [30]. We therefore investigated the impact of intravenous infection of 02363-EG and 02363-KE on lymphocyte subsets in infected monkeys. Peripheral blood was collected at 3, 7, and 10 days p.i. from each monkey and analyzed by flow cytometry to detect lymphocyte subsets: CD3^+^CD4^+^ (CD4^+^ T cell), CD3^+^CD8^+^ (CD8^+^ T cell), CD3^-^CD16^+^ (NK cell), and CD3^-^CD20^+^ (B lymphocyte) cells (Fig 3 and S2 Fig). The average pre-inoculation number of lymphocytes in PBMC from eight monkeys in both groups was used as an uninfected control (Fig 3, Day 0). In four 02363-KE-inoculated monkeys, lymphocyte numbers decreased overall at 3 days p.i., and then recovered in later stages of infection across all lymphocyte subsets demonstrating a transient lymphocytopenia at 3 days p.i. A significant difference in the number of CD3^-^CD16^+^ cells was observed between 02363-KE-inoculated and uninfected control groups 3 days p.i. (Fig 3C) and a trend towards decreased numbers of CD3^+^CD4^+^ (*P* = 0.08, Fig 3A) CD3^+^CD8^+^ (*P* = 0.07, Fig 3B), and CD3^-^CD20^+^ (*P* = 0.09, Fig 3D), was observed in the KE-inoculated group compared to the uninfected group 3 days p.i. The number of CD3^-^CD16^+^ (Fig 3C) and CD3^-^CD20^+^ (Fig 3D) cells increased at 7 days p.i. in the 02363-KE-inoculated monkeys with a significant difference compared to uninfected controls.

In contrast, the 02363-EG-infected group demonstrated almost entirely stable lymphocyte kinetics (Fig 3 and S2 Fig), indicating no apparent lymphocytopenia with the exception of monkey #5134 which exhibited a decline in CD3^+^CD4^+^, CD3^+^CD8^+^, and CD3^-^CD20^+^ lymphocytes in the later stages of infection, particularly at 10 days p.i. (Fig 3A, 3B, and 3D, right panel). Thus, transient lymphocytopenia was observed apparently in the 02363-KE-infected group during the early stages of infection but not in the 02363-EG-infected group with the exception of monkey #5134 in the later stages of infection.

### Induction of circulating cytokines

**Fig 4:**
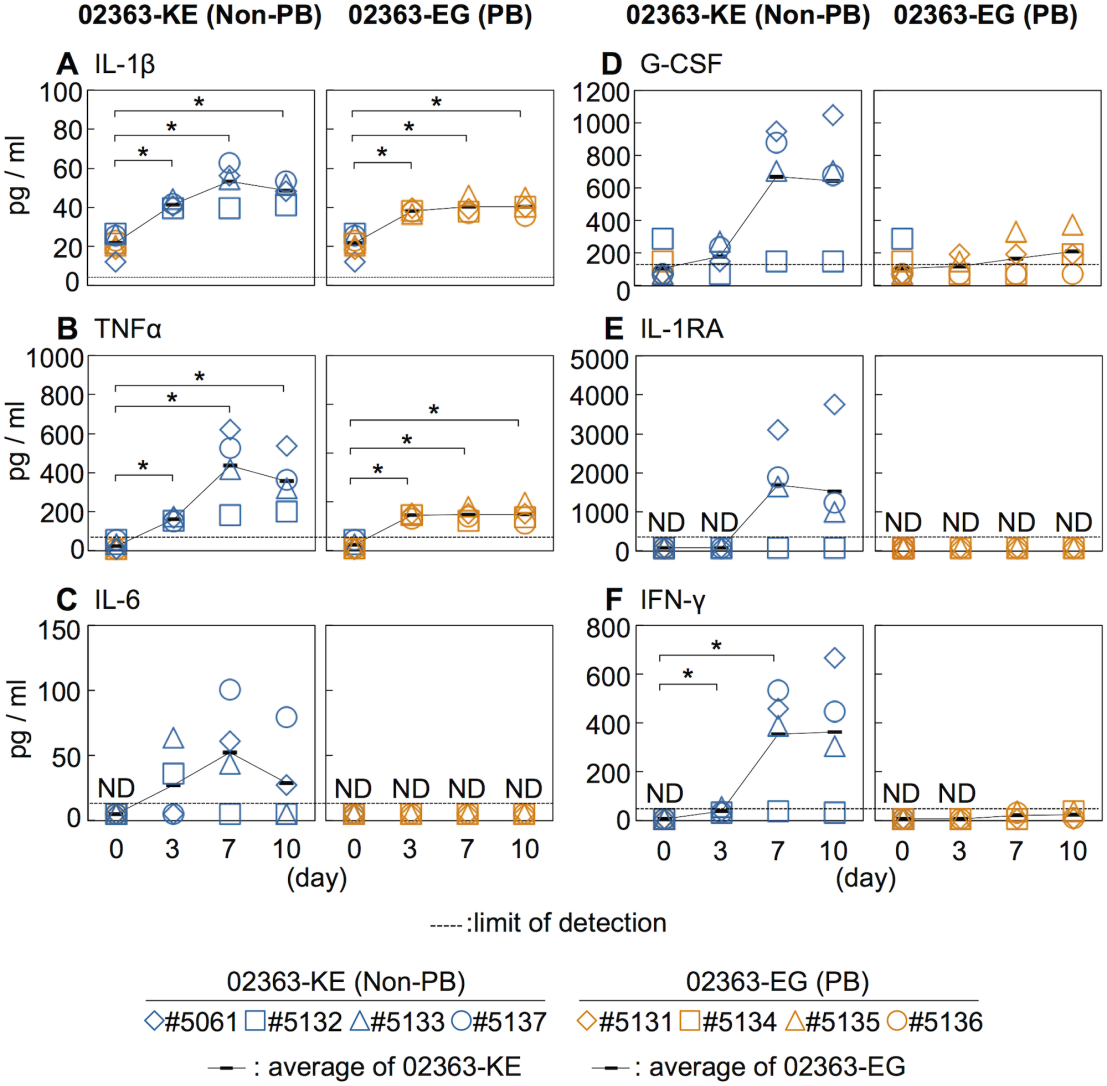
Serum cytokine level of the inoculated monkeys. Serum levels of cytokines, (A) IL-1β, (B) TNFα, (C) IL-6, (D) G-CSF, (E) IL-1RA, and (F) IFN-γ collected from the monkeys inoculated with EV71-02363-KE (non-PB) or EV71-02363-EG (PB) were analyzed using Luminex 200. Cytokine levels of all eight preinoculation monkeys are shown at Day 0 and the average was used as the uninfected control. Serum cytokine levels, collected at 3, 7, and 10 days postinfection from the 02363-KE-inoculated group are indicated in blue and those from the 02363-EG-inoculated group was indicated in orange. ND (not detected) indicates that serum cytokine levels of all samples are below the limit of detection (dashed lines). Average of cytokine concentrations are indicated as black solid line plot. Significant differences between the preinoculation control (eight monkeys at Day 0) and postinfection (four monkeys) at indicated days were determined using Student’s *t*-test and *P* values < 0.05 are marked by asterisks. Significant differences in cytokine levels between 02363-KE- and 02363-EG-inoculated groups at indicated days are shown in supplemental S3 Fig.

Increased serum levels of pro-inflammatory cytokines, IFN-γ, IL-6, and TNFα are observed in patients with EV71-associated encephalitis and pulmonary edema compared to patients with uncomplicated HFMD [4, 45–48]. Moreover, increased serum levels of other cytokines, including IL-1β, IL-1RA, and G-CSF, are associated with poor prognosis in EV71 infection and therefore considered possible markers of severe EV71 infections in humans [4, 45–48]. We measured the serum cytokine levels during EV71 infection to investigate the immune response following inoculation with 02363-EG and 02363-KE strains. The average pre-inoculation serum cytokine level of all eight monkeys was used as an uninfected control (Fig 4, Day 0). As shown in Fig 4A and 4B, and S3 Fig, a significant increase in serum levels of the pro-inflammatory cytokines IL-1β and TNF-α was observed in both groups 3–10 days p.i. A non-significant increase in serum IL-6 levels compared to control levels was observed in 02363-KE-inoculated monkeys but not in 02363-EG-inoculated monkeys (Fig 4C and S3 Fig). Increased levels of other cytokines (G-CSF, IL-1RA, and IFN-γ) were also observed, particularly IFN-γ, in the 02363-KE-inoculated monkeys except #5132 at 7–10 days p.i., but not in the EG-inoculated monkeys (Figs 4D–4F). In general, cytokine responses were more evident in 02363-KE-inoculated monkeys than those in EG-inoculated monkeys.

### Virus detection and isolation from clinical and tissue samples

To monitor viral spread following intravenous inoculation with 02363-EG and 02363-KE strains, and identify primary and secondary sites for viral replication, clinical samples (throat swabs, rectal swabs, serum, and PBMC) were collected at 3, 7, and 10 days p.i. and autopsy samples obtained at 10 days p.i. were used for inoculation of RD cells. Almost all cytopathic effects (CPEs) appeared during the second blind passage following inoculation of RD cells except for a spleen sample from #5134 where CPE was observed after the first inoculation (10^4.1^ CCID_50_/g tissue). As viral titers in clinical and autopsy samples were generally low, we also performed direct detection of viral RNA from clinical and tissue samples. The sensitivity of consensus-degenerate hybrid oligonucleotide primer (CODEHOP) RT-PCR (semi-nested RT-PCR for enteroviruses) [49] is higher than that for virus isolation using RD cells. All samples found to be positive by viral isolation were found to be positive by molecular detection and a number of samples found to be negative by viral isolation were found to be positive only by molecular detection (denoted by “+” and “±,” respectively, in Tables 1 and 2).

**Table 1:** Amino acid residues at VP1-98 and VP1-145 of EV71 variants in clinical samples.

		02363-KE (non-PB)–inoculated monkeys								02363-EG (PB)–inoculated monkeys							
Clinical samples	Days p.i.	#5061		#5132		#5133		#5137		#5131		#5134		#5135		#5136	
Rectal swab	3	++	KE	++	KE/**E**E	+	**E**E	++	KE	−		−		−		−	
	7	+	KE	−		−		++	KE	−		−		−		−	
	10	−		++	KE	++	**E**E	++	**E**E	−		+	E**E**	−		−	
Throat swab	3	++	KE/**E**E	++	KE/**E**E	++	**E**E	++	KE	−		−		+	n.d.	−	
	7	+	**E**E	+	**E**E	++	**E**E	+	KE	−		−		−		−	
	10	−		+	**E**E	+	KE, K242**Q**	−		−		+	n.d.	−		−	
Serum	3	++	**E**E	+	**E**E	++	**E**E	+	**E**E	−		−		−		+	E**E**
	7	+	n.d.	−		−		−		−		+	E**E**	+	E**E** *	±	n.d.
	10	+	n.d.	−		−		−		−		+	E**E**	±	n.d.	±	n.d.
PBMC	3	+	**E**E*	+	**E**E*	+	**E**E/KE*	+	**E**E*	±	n.d.	−		±	n.d.	±	n.d.
	7	−		−		−		−		±	n.d.	±	n.d.	±	n.d.	±	n.d.
	10	−		−		−		−		±	n.d.	+	E**E** *	+	EG*	+	EG*

++; Positive by both virus isolation and molecular detection.

+; Virus isolation negative but positive by molecular detection.

±; Virus isolation negative but positive by molecular detection with a weak signal.

-; Negative by both virus isolation and molecular detection.

“KE” represents deduced amino acid residues at VP1-98**K** and VP1-145**E**. “KE/EE” indicates mixed nucleotides [K(AAA) and E(GAA)] were identified at VP1-98 on sequencing chromatograms obtained from clinical samples by direct RT-PCR. n.d.; sequences could not be determined.

*Sequence results were determined from the partial VP1 region obtained by the CODEHOP RT-PCR. Amino acid substitutions from the original inoculated viruses are indicated in bold. For amino acid residues in the VP1 region examined, amino acid substitutions were identified only at VP1-98 and VP1-145, except a substitution from VP1-242K to 242Q in a throat swab sample from monkey #5133 at 10 days postinfection.

**Table 2:** Amino acid residues at VP1-98 and VP1-145 of EV71 variants in postmortem tissue samples.

		02363-KE (non-PB)–inoculated monkeys								02363-EG (PB)–inoculated monkeys							
Organ	Tissue (10 days p.i.)	#5061		#5132		#5133		#5137		#5131		#5134		#5135		#5136	
CNS	Lumber code	+	KE/**E**E	±	n.d.	+	KE/**E**E, K242**Q**	+	KE/**E**E, K242**T**	−		+	E**E** *	+	E**E**	+	E**E**
	Cervical code	−		++	**E**E	±	n.d.	+	**Q**E/**E**E	−		++	E**E**	++	E**E**	++	E**E**
	Medulla oblongata	±	n.d.	±	n.d.	±	n.d.	±	n.d.	−		−		++	E**E**	++	E**E**
	Midbrain	−		−		±	n.d.	±	n.d.	−		−		+	E**E**	−	
	Cerebrum	−		−		−		−		−		−		++	E**E**	++	E**E**
	Cerebellum	±	n.d.	−		−		±	n.d.	±	n.d.	−		−		±	n.d.
	Pons	−		−		−		−		−		−		+	E**E**	+	E**E**
Main organ	Lung	−		−		−		−		−		−		−		−	
	Heart	−		−		++	**Q**E/**E**E	±	n.d.	−		−		−		−	
	Liver	−		−		-		-		−		+	E**E**	−		−	
	Spleen	−		−		-		-		−		++	E**E**	−		−	
	Kidney	−		++	**Q**E/**E**E	+	KE, K242**Q**	+	**E**E	−		+	E**E**	−		−	
Lymph node	Superficial cervical	+	**E**E	+	KE, K242**E**	+	KE	+	KE	±	n.d.	+	E**E**	+	E**E**	+	E**E**
	Deep cervical	+	KE	+	**E**E, A58**S**	++	**E**E	+	**E**E	±	n.d.	++	E**E**	±	n.d.	±	n.d.
	Mesentery	−		−		−		−		−		+	E**E**	±	n.d.	±	n.d.
	Tonsil	−		−		−		−		−		++	E**E**	−		−	
	Peyer’s patch	−		−		−		−		−		++	E**E**	−		−	
Other	Dorsal root ganglion	−		+	**E**E*	++	**E**E	+	**E**E*	−		++	E**E**	−		−	
	Trigeminal nerve	−		+	**E**E*	+	**E**E*	−		−		+	E**E** *, L95**I**	−		−	
	CSF	−		−		−		−		−		−		−		−	

++; Positive by both virus isolation and molecular detection.

+; Virus isolation negative but positive by molecular detection.

±; Virus isolation negative but positive by molecular detection with a weak signal.

−; Negative for both virus isolation and molecular detection.

“KE” represents deduced amino acid residues at VP1-98**K** and VP1-145**E**. “KE/EE” indicates that mixed nucleotides [K(AAA) and E(GAA)] were identified at VP1-98 on sequencing chromatogram obtained from the clinical samples by direct RT-PCR. n.d.; the sequences could not be determined.

*The sequence results were determined from the partial VP1 region obtained by the CODEHOP RT-PCR. Amino acid substitutions from the original inoculated viruses are indicated in bold. Amino acid substitutions other than VP1-98 and VP1-145 in the entire or partial VP1 sequence were indicated as K242Q, or K242T, etc.

**Fig 5:**
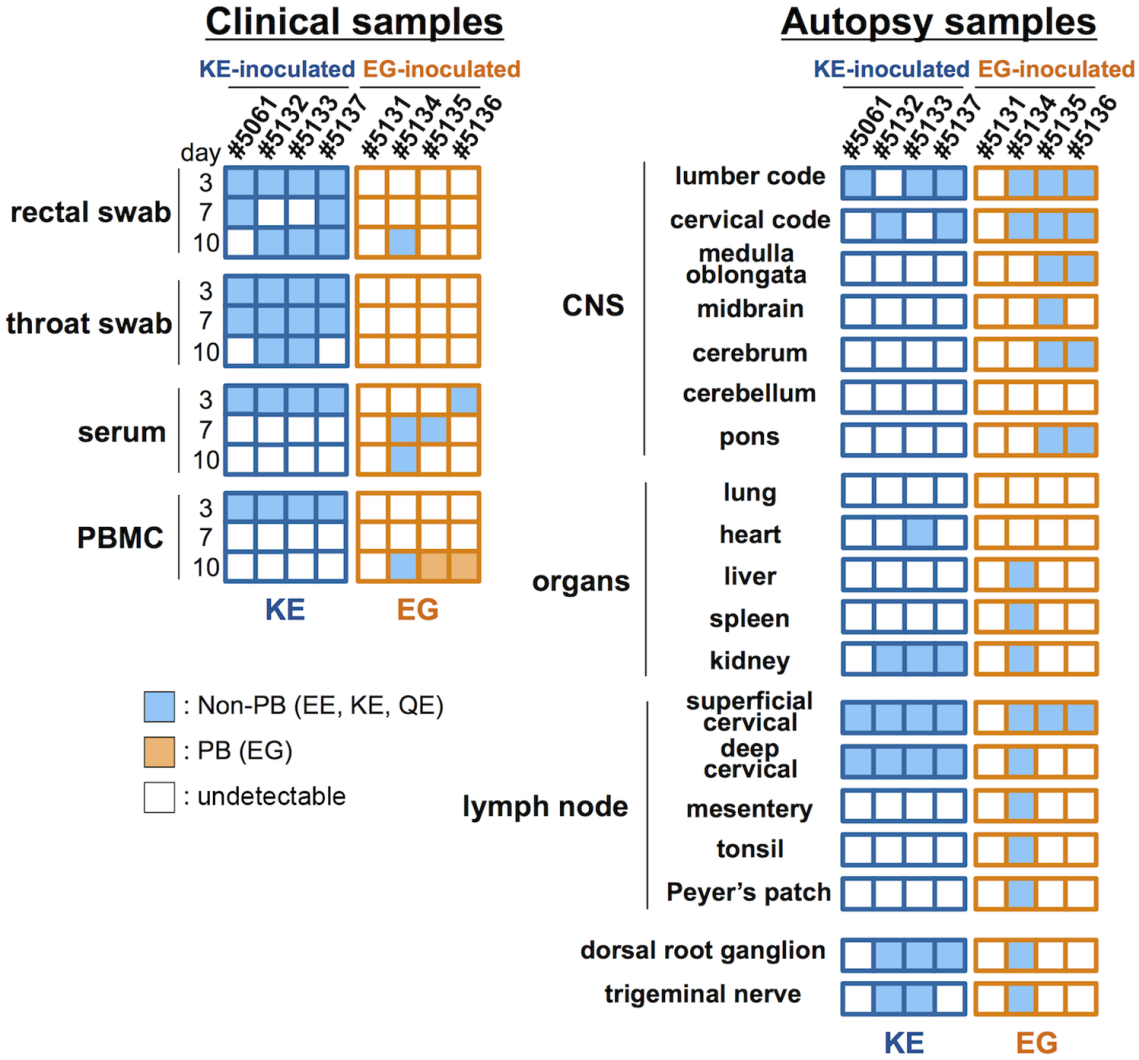
Schematic illustration of the detection of non-PB and PB variants of EV71. Virus detection and genetic variants of EV71 from clinical samples (Table 1) and postmortem tissue samples (Table 2) are summarized. Positive samples by molecular detection (++ or + in Tables 1 and 2) for EV71 are indicated and classified into PB (VP1-145G) or non-PB (VP1-145E) variants according to the amino acid residue at VP1-145 from the partial VP1 sequences determined by direct RT-PCR from samples. PB and non-PB variants are shaded in orange and blue, respectively.

As shown in Table 1 and summarized in Fig 5, EV71 was detected in throat swabs, rectal swabs, serum samples, and PBMC samples from all four 02363-KE-inoculated monkeys at 3 days p.i., demonstrating efficient induction of acute viremia. In 02363-KE-inoculated monkeys, EV71 was detected in some throat and rectal swab samples, but not in serum and PBMC samples, in the later stages of infection at 7 and 10 days p.i. On the other hand, EV71 was rarely detected in throat and rectal swab samples from 02363-EG-inoculated monkeys throughout the experimental period but was detected in some serum samples mainly in the later stages of infection, except in monkey #5131. EV71 was detected in some PBMC samples of 02363-EG-inoculated monkeys during the early stages of infection with a faint signal (denoted by “±”); however, three out of four 02363-EG-inoculated monkeys had apparently CODEHOP-RT-PCR-positive PBMC samples at 10 days p.i. In general, EV71 was frequently detected in various clinical samples from 02363-KE-inoculated monkeys during the period of acute viremia.

At 10 days p.i., EV71 was detected in some central nervous system (CNS) and non-CNS postmortem tissue samples in both groups, except monkey #5131 (Table 2, Fig 5). In general, EV71 was identified at a higher frequency in the lumbar and cervical spinal cord samples in CNS tissues and in cervical lymph nodes in non-CNS tissues in both groups (Table 2). In 02363-KE-inoculated monkeys, EV71 was also detected in kidney (3/4 monkeys), dorsal root ganglion (3/4 monkeys), and trigeminal nerve (2/4 monkeys) samples. In one 02363-EG-inoculated monkey #5134, EV71 was detected mainly in non-CNS tissues, serum, and PBMC samples, suggesting a viremia peak at 10 days p.i. On the other hand, in two 02363-EG-inoculated monkeys (#5135 and #5136), EV71 was extensively detected in CNS tissues, including medulla oblongata, cerebrum, and pons (Table 2 and Fig 5).

### *In vivo* genetic stability of PB and non-PB strains

To investigate the genetic and phenotypic stabilities of PB and non-PB strains of EV71 following inoculation into monkeys, we determined the partial and/or entire VP1 sequences, which are mainly responsible for the PSGL-1-binding phenotype. To minimize selection and adaptation bias of EV71 variants during cell culture, we attempted to amplify the entire VP1 region of EV71 directly from the clinical and autopsy samples by RT-PCR without virus isolation (Tables 1 and 2, Fig 5). When entire VP1 fragments could not be amplified from samples by RT-PCR, we applied a highly sensitive CODEHOP RT-PCR to amplify the partial VP1 region (approximately from VP1-45 to VP1-162), including amino acid residues at VP1-98 and VP1-145.

As shown in Table 1, amino acid combinations at VP1-98 and VP1-145, identified from clinical samples of 02363-KE-inoculated monkeys were VP1-98K and VP1-145E (KE) or VP1-98E and VP1-145E (EE). The EE variants of EV71 were detected in serum and PBMC samples from all four 02363-KE-inoculated monkeys at 3 days p.i. (Table 1), suggesting rapid amino acid substitution of VP1-98K by VP1-98E following 02363-KE-inoculation (Fig 5). The EE variants of EV71 were frequently detected in throat and rectal samples throughout the experimental period, and also detected in CNS and non-CNS tissues at 10 days p.i. (Table 2). In postmortem tissues (Table 1), VP1-98 was heterogeneous (K, E, or Q) at VP1-98 and viral quasi-species was observed in a number of clinical samples (Table 1 and S1 Table). However, the major determinant of the PSGL-1-nonbinding phenotype, VP1-145E, was consistently stable in the 02363-KE-inoculated monkeys, suggesting that the original KE strain and EE variants, both with a non-PB phenotype, efficiently induced acute viremia with further spread to the CNS tissues, and excretion in throat and stool samples in 02363-KE-inoculated monkeys, presumably by a PSGL-1-independent mechanism.

Although there was a low detection rate in clinical samples from 02363-EG-inoculated monkeys, EE variants with a VP1-G145E substitution were detected in several serum samples from three monkeys (Table 1). The EG strain was not identified in any swab or serum samples from any of the four monkeys during the experimental period. However, EG variants were identified in PBMC samples in two 02363-EG-inoculated monkeys (#5135 and #5136) by CODEHOP RT-PCR (Table 1, Fig 5). In 02363-EG-inoculated monkeys, all EV71 variants identified from the CNS tissues possessed a VP1-G145E substitution (EE variant, Table 2). EE variants were also detected in superficial cervical lymph nodes (3/4 monkeys) and extensively detected in various non-CNS tissues in one 02363-EG-infected monkey (#5134). No EG strains were detected in CNS or non-CNS tissues in the 02363-EG-infected monkeys, revealing a strong mutation/selection bias from VP1-145G to VP1-145E during the *in vivo* replication of the original 02363-EG strain in cynomolgus monkeys. Thus, different phenotypic variants of EV71 (EE and EG variants) were detected in both monkeys #5135 and #5136 at 10 days p.i. (Fig 5). No detectable amino acid substitutions or viral quasi-species were identified at VP1-98E in clinical or tissue samples from EG-infected monkeys (Tables 1 and 2, Fig 5, and S1 Table).

With the exception of two amino acids, VP1-98 in the 02363-KE-infected monkeys and VP1-145 in 02363-EG-infected monkeys, no common amino acid substitutions were identified in the VP1 region among EV71 variants from clinical and tissue samples from both groups (Tables 1 and 2). However, in some tissue and clinical samples from three 02363-KE-infected monkeys, unique amino acid substitutions (K242Q, K242T, and K242E) were found at a conserved lysine residue at VP1-242, which was identified as a minor determinant of PSGL-1-binding phenotype in combination with VP1-145G [30]. Other amino acid substitutions were found at A58S and L95I in the VP1 region of EV71; however, amino acid residues in the VP1 region are generally stable except three unique amino acids at VP1-98, VP1-145, and VP1-242, during *in vivo* replication of EV71 in monkeys.

### EV71 variants in tissue and clinical samples

To analyze viral quasi-species with variable amino acid residues at VP1-98 and VP1-145 in EV71 variants from clinical and tissue samples, partial viral cDNA was amplified directly from the samples by RT-PCR and cloned into bacterial plasmid vectors. Independent plasmid clones were then sequenced.

In four clinical samples from the 02363-KE-inoculated monkeys, VP1-98 was highly heterogenous (K, Q, N, or E), suggesting high quasi-species generation and low selection at VP1-98, but VP1-145E was identical in 30 independent clones (S1 Table). Among six tissues samples examined (two from 02363-KE-inoculated and four from 02363-EG-inoculated monkeys), which were identified as EE variants by direct VP1 sequencing, all of the 44 plasmid clones were found to possess VP1-98E (GAA) and VP1-145E (GAG) sequences, representing no apparent quasi-species variants (S1 Table). As expected, in a PBMC sample from a 02363-EG-inoculated monkey, #5136, (10 days p.i.), an amino acid residue at VP1-145 was heterogenous and VP1-145G was dominant (eight out of nine clones). The results suggest that the rate of quasi-species generation and selection at VP1-98 and VP1-145 was varied in different clinical and tissue samples.

### Histological analysis of CNS tissues and neurovirulence of EV71 variants

**Fig 6:**
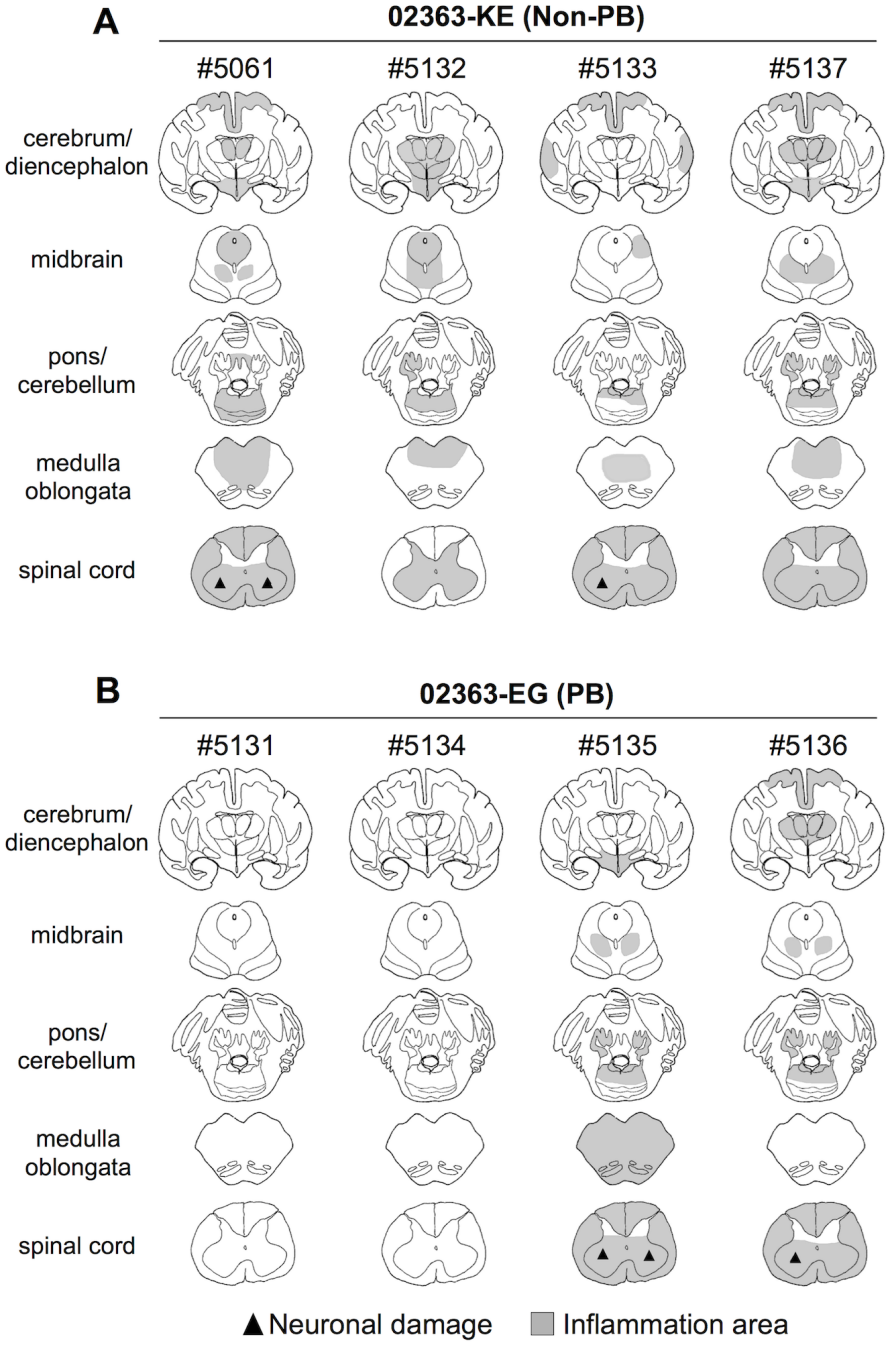
Distribution of EV71-induced lesions in CNS tissues. Postmortem CNS tissues at 10 days postinfection from four monkeys inoculated with either (A) EV71-02363-KE (non-PB) or (B) EV71-02363-EG (PB) strain. Rows, from top to bottom, indicate the cerebrum/diencephalon, midbrain, pons/cerebellum, medulla oblongata and spinal cord, respectively. Filled triangle indicates neuronal damage and the gray areas show inflammation in the parenchyma and the meninges of the central nervous system.

The CNS tissue samples from the 02363-KE- or 02363-EG-inoculated monkeys were histopathologically examined to compare the neuropathogenesis of EV71 strains. Inflammation and neuronal degeneration were extensively found in different CNS tissues from all four 02363-KE-inoculated monkeys at 10 days p.i. (Fig 6A). Inflammation in the spinal cord and gray matter of cerebrum (Fig 6A) and the induction of lymphocytes in cerebrospinal fluid (CSF) samples (S2 Table, S4 Fig) indicate myelitis, encephalitis, and meningitis occurred in three monkeys, #5061, #5133, and #5137 at 10 days p.i. Neuronal degeneration was observed in the spinal cord anterior horn of two monkeys, #5061 and #5133 (Fig 6A). Inflammation was observed in the brainstem of one monkey #5132. Restriction of inflammation to the spinal cord gray matter and a decline the number of lymphocytes present in CSF samples between 7 days p.i. and 10 days p.i., suggested monkey #5132 might have a peak in meningoencephalitis at 7 days p.i., and was presumably within the convalescent phase at 10 days p.i. (Fig 6A and S2 Table).

**Fig 7:**
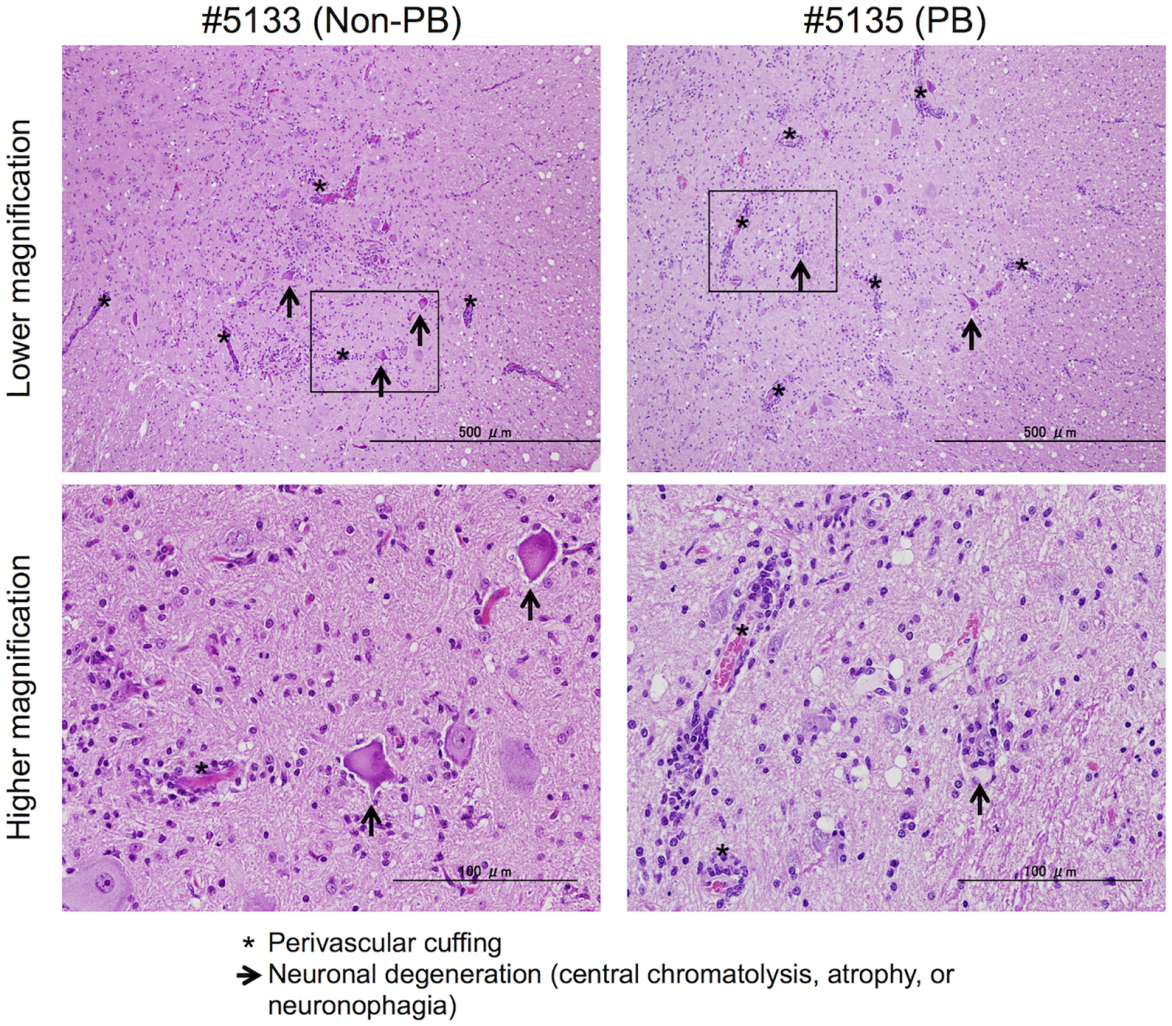
Histopathological spinal cord findings. Representative spinal cord sections from monkeys inoculated with EV71-02363-KE (non-PB; #5133) or EV71-02363-EG (PB; #5135) strain, stained with hematoxylin and eosin (H&E). Typical histopathological changes are shown in the spinal cord from both samples. Upper panels: Prominent inflammatory infiltrations including mononuclear cuffing (*) and gliosis were seen in the anterior horn of the spinal cords of both monkeys. Neuronal degeneration (arrows) was observed within the lesion. Scale bar; 500 μm. Lower panels: perivascular cuffing with mononuclear cells was observed within the lesion (*) with higher magnification of the squared area of upper panel. Lower left panel: Loss of nissle granules within some motor neurons (central chromatolysis, arrow). Lower right panel: neuronophagia observed within the lesion (arrow). Scale bar; 100 μm.

Inflammation and neuronal degeneration were observed in two 02363-EG-inoculated monkeys, #5135 and #5136. Although lymphocytes were failed to investigate in some CSF samples due to blood contamination (S2 Table), these monkeys had histopathological evidence of myelitis, encephalitis, and meningitis at 10 days p.i. (Fig 6B). However, other two monkeys, #5131 and #5134, had no apparent CNS lesions (Fig 6B). Histopathological results in 02363-EG-inoculated monkeys were mostly consistent with the distribution of viral infection, shown in Table 2. There was no significant difference in typical neuronal lesions of the spinal cord between 02363-KE- and 02363-EG-inoculated monkeys; there was mild neurodegeneration with perivascular mononuclear cuffing and central chromatolysis and gliosis of the anterior horn of the spinal cord in both groups (Fig 7). Taken together with virus detection (Table 2) and histopathological (Fig 6) analyses of the CNS tissues, all four KE-inoculated monkeys were likely in the late or convalescent stages of meningoencephalitis at 10 days p.i. Two out of four 02363-EG-inoculated monkeys (#5135 and #5136) demonstrated a peak of meningoencephalitis and monkey #5134 developed viremia without apparent CNS symptoms at 10 days p.i. In conclusion, histopathological analysis of CNS tissues at 10 days postinfection revealed that 02363-KE induced neuropathogenesis (inflammation and neuronal damage) more efficiently than that induced by 02363-EG.

## Discussion

To elucidate the *in vivo* effects of PSGL-1-dependent replication and pathogenesis, in this study, we used a cynomolgus monkey model to assess viral replication, pathogenicity, and the genetic stability of PB and non-PB strains of EV71. Mouse models have been extensively used to study the pathogenesis of EV71 infection. However, amino acid identity between human and murine PSGL-1 molecules is not high, particularly in the N-terminal region of PSGL-1, which is responsible for the binding of human PSGL-1 to EV71. Accordingly, a PB strain of EV71-1095 was shown not to bind to mouse PSGL-1 [16]. Therefore, to investigate PSGL-1-dependent viral replication and pathogenesis, mouse models would be unreliable as the PB strains of EV71 may not replicate in mice in a PSGL-1-dependent manner. On the other hand, the amino acid identity of the EV71-binding region of PSGL-1 (amino acids 42–61; QATEYEYLDYDFLPETEPPE) between human and cynomolgus monkey PSGL-1 molecules is 100%, thus indicating that a monkey model, more closely resembled the human, would be more reliable than mouse models in investigating PSGL-1-dependent EV71 replication. In our previous studies, cynomolgus monkeys infected *via* intravenous inoculation with EV71 demonstrated similar neurological manifestations to those observed in humans including paralysis, ataxia, tremor, and development of CNS lesions in the cerebrum, medulla oblongata, and spinal cord [42–44]. In addition, this monkey model allows the identification of EV71 variants in various clinical and tissue samples, including CNS and non-CNS tissues, to monitor viral spread and genetic stability of EV71 in infected individuals in more detail than smaller mouse models.

After the intravenous inoculation with the 02363-KE (non-PB) strain, KE or EE variants were detected in clinical samples from different locations (throat and rectal swabs, serum, and PBMC) in all four monkeys at 3 days p.i., demonstrated acute viremia associated with transient lymphocytopenia (Fig 3) and cytokine induction (Fig 4) in the early stage of infection. In the later stages of infection at 10 days p.i., KE or EE variants were detected from lumbar and cervical spinal cord samples of 02363-KE-inoculated monkeys (Fig 5) that developed typical neuropathogenesis (inflammation and neuronal damage) (Fig 6). Thus, the KE or EE variants efficiently induced viremia and CNS involvement with apparent neurological manifestations (Fig 2), presumably in a PSGL-1-independent manner as a major determinant of the non-PB phenotype, VP1-145E, was genetically stable during the *in vivo* replication and spread to CNS and non-CNS tissues in 02363-KE-inoculated monkeys (Fig 5, Table 2, and S1 Table).

The emergence of EE variants with a substitution of glycine from glutamic acid at VP1-145 in serum samples (Table 1) and their wide distribution throughout CNS and non-CNS tissues in 02363-EG-infected monkeys (Table 2) again suggested EE variants may confer better *in vivo* fitness than the inoculated EG strain during the acute viremia phase. Following viremia, resultant EE variants were mainly responsible for the CNS involvement in monkeys #5135 and #5136 in a PSGL-1-independent manner. SCARB2 was histopathologically identified in a number of CNS and non-CNS tissues, including EV71 antigen-positive neuronal cells in human encephalitis cases [50], and neuronal cells in EV71-infected SCARB2-transgenic mice [25]. Therefore, SCARB2-expressing neuronal cells in the CNS may play a critical role in direct CNS involvement during EV71 infection in a PSGL-1-independent manner [13, 51].

It is likely that some time is required for mutation and selection of EE variants following 02363-EG-inoculation, possibly resulting in slower development of pathogenesis in the 02363-EG-inoculated group than the 02363-KE-inoculated group. Mutation and selection of EE variants may also contribute to individual variability in the pathogenesis observed in the four EG-inoculated monkeys at 10 days p.i.; inefficient infection in monkey #5131, acute viremia in monkey #5134, and meningoencephalitis in monkeys #5135 and #5136 (Figs 5 and 6). In monkey #5134, EE variants were detected in some clinical samples, various non-CNS tissues, and some CNS tissues (lumbar and cervical spinal cord) only in the later stages of infection (Tables 1 and 2). At the same time, this monkey developed typical lymphocytopenia at 10 days p.i. (Fig 3), observed in four 02363-KE-inoculated monkeys at 3 days p.i., consistent with slower development of viremia in monkey #5134 than in 02363-KE-inoculated monkeys.

EG variants were detected in PBMC samples from two 02363-EG-inoculated monkeys (#5135 and #5136) during the meningoencephalitis phase at 10 days p.i. (Table 1, Fig 5) indicating possible involvement of PSGL-1-dependent EV71 replication in local, specific immune cell types. Viral quasi-species variants with VP1-145G and VP1-145E were identified in a PBMC sample from monkey #5136 (S1 Table). It is uncertain whether the inoculated 02363-EG strain was survived in minor PSGL-1-positive immune cells without a VP1-G145E substitution or EG variants were further mutated from circulating EE variants by acquiring viral fitness in the local cell microenvironments in the later stages of infection. We cannot exclude the possibility that additional mutations outside of the capsid VP1 region may compensate for the *in vivo* fitness of EG variants. However, we were unable to isolate infectious EG variants in cell culture, and detected viral RNA only by RT-PCR directly from PBMC samples, presumably due to low virus titers in the samples (Table 1).

Recent studies have demonstrated that rhesus monkeys exhibit neuropathogenesis following the EV71 infection [52] and CD14-positive lymphocytes in peripheral blood are responsible for the efficient EV71 replication [53]. Therefore, to confirm replication competency of 02363-EG and 02363-KE strains in monkey PBMCs and CD14-positive lymphocytes, we isolated PBMCs and CD14-positive lymphocytes from four healthy monkeys and infected them with the EV71 strains (1 CCID_50_/cell). No apparent virus replication was observed in the monkey PBMCs or CD14-positive lymphocytes with either of the strains (S5 Fig) in-keeping with results from our previous study using human PBMCs [54]. Thus, we have yet to identify the specific immune cell population in peripheral blood responsible for PSGL-1-dependent replication of EV71.

In this study, we demonstrated potent *in vivo* selection of VP1-145E variants in a cynomolgus monkey model. Similar *in vivo* mutation and selection from G/Q to E at VP1-145 has been reported in several mouse models using mouse-adapted EV71 strains [34–37] and VP1-145E has been identified as a critical virulence determinant in mice alone or in combination with other amino acids. As mentioned above regarding the structural differences between human and mouse PSGL-1, it is unlikely that a phenotypic change from PB to non-PB by VP1-145 substitution also contributes to the improved mouse PSGL-1-binding of VP1-145E mutants. Instead, VP1-145E may be responsible for increased virulence in mice thorough unknown mechanisms, such as the involvement of the other receptors and/or cellular proteins in viral entry, uncoating, or host immune responses to EV71, regardless of the PSGL-1-binding capability of EV71. We speculate the same mechanism underlies *in vivo* fitness and virulence associated with VP1-145E in both mouse and monkey models, however, the viral and cellular factors determining *in vivo* phenotypes of EV71 have yet to be elucidated.

One possible explanation for the rapid selection of EV71 variants with amino acid substitutions at VP1-98 or VP1-145 is that escape mutants were selected under immunological pressure as VP1-98 and VP1-145 are located at possible neutralizing epitopes of EV71 [55]. However, the EE variants with substitution of K by E at VP1-98 were frequently identified in the serum samples from 02363-KE-inoculated monkeys, even at 3 days p.i. before the induction of neutralizing antibodies (Table 1, S1 Fig). Moreover, cross-neutralizing antibodies against both strains were induced in both groups (S6 Fig), suggesting neutralization escape is not the most critical factor for rapid selection of EV71 variants in this monkey model.

We have not established an efficient oral infection model in non-human primates [56]; therefore, the route of virus inoculation in this study (intravenous inoculation) does not reflect the natural EV71 infection in humans. After intravenous inoculation with 02363-KE, following viremia, infectious KE and EE variants were excreted in throat and rectal swab samples. EE variants are commonest among EV71 isolates available in GenBank; therefore, excreted EE variants in the monkeys may be transmissible. However, we cannot exclude the possible involvement of PSGL-1-dependent replication following the primary mucosal infection of EV71 as various PSGL-1-expressing leukocytes are located in pharyngeal and intestinal mucosal tissues [27, 57]. In this monkey model several typical clinical manifestations associated with EV71 infections in humans, including HFMD-like skin and oral rashes (mild local inflammation), polio-like paralysis, and fatal pulmonary edema (more severe clinical manifestations of CNS disease), were not observed. Moreover, we have not investigated the *in vivo* phenotypes of other PB strains such as the 02363-EQ strain [30]. Thus, we were unable to investigate all aspects of PSGL-1-dependent and -independent pathogenesis in EV71 infections in this cynomolgus monkey model.

To our knowledge, this is the first comprehensive study to describe the evolutionary dynamics and neuropathogenesis of EV71 within infected individuals in a non-human primate model. In a recent study, Cordey *et al*. reported virus detection and characterization in several different clinical samples from an EV71-infected immunocompromised patient and identified an amino acid at VP1-97 as a potential determinant of host adaptation and neurovirulence in humans by comparing full-length genome sequences and *in vitro* phenotypes of EV71 variants [58]. In the present study, we used cDNA-derived EV71 strains to minimize *in vitro* quasi-species and examined the dynamics of viral distribution in clinical and tissue samples, genetic stabilities, viral fitness, and the neuropathogenesis in various CNS tissues in EV71-infected monkeys. We identified two amino acids in the VP1 region, VP1-98 and VP1-145, as variable sites during the *in vivo* viral replication in the monkeys, which may be associated with the apparent amino acid polymorphism observed among the field EV71 isolates. Moreover, unique amino acid substitutions were identified at VP1-242 in some clinical and tissue samples from 02363-KE-inoculated monkeys (Tables 1 and 2). Among them, VP1-145 is a major determinant of neuroinvasion to the CNS and neuropathogenesis as all EV71 variants detected in CNS tissues consistently possessed VP1-145E in both 02363-KE- and 02363-EG-inoculated groups (Table 2 and Fig 5). On the other hand, amino acid residues among EV71 variants from CNS tissues were variable and heterogenous at VP1-98 (K, E, or Q) and VP1-242 (K, Q, or T) (Tables 1 and 2, S1 Table).

Recent structural analyses of EV71 have revealed VP1-98, VP1-145, and VP1-242 are located within surface loops (BC, DE, and HI, respectively) surrounding the center of the 5-fold mesa of EV71 virions [2, 59]. These three amino acid residues have previously been identified as critical determinants of several distinct EV71 phenotypes, including PSGL-1-binding (VP1-145 and VP1-242) [30], putative heparan sulfate-binding (VP1-242) [17], neutralizing antibody-binding (VP1-98, VP1-145, and VP1-242) [55], adaptation and virulence in mouse models (VP1-145) [34–37], and positive selection in humans (VP1-98 and VP1-145) [31–33]. In addition, other amino acids, adjacent to these three amino acids, are also involved in certain EV71 phenotypes alone or in combination with these three amino acids; VP1-97 as a possible virulence determinant in humans [58], VP1-243 as a cyclophilin A-binding site [60], and VP1-244 as a determinant of PSGL-1-binding [30] and mouse adaptation/virulence [61]. A growing number of molecular epidemiological studies have demonstrated that some of the critical residues in the VP1 region are variable (VP1-98K/E/Q/N/G and VP1-145E/G/Q/A/K/R) but that others are apparently conserved (VP1-97L, VP-242K, VP1-243S, and VP1-244K) among EV71 isolates. Recent studies have also suggested VP1-145G/Q isolates were more frequently detected from cases with severe neurological disease in humans than VP1-145E isolates [38–41]. As we have demonstrated for the PSGL-1-binding phenotype [30], combinations and mutations in several critical amino acid residues, including VP1-98, VP1-145, and VP1-242, may be responsible for the distinct *in vitro* and *in vivo* phenotypes of EV71 and possibly associated with the overall pathogenesis in humans.

In this study, we examined the *in vivo* evolutionary dynamics and neuropathogenesis of PB and non-PB strains of EV71 in a non-human primate model. We demonstrated potent *in vivo* selection of VP1-145E variants. VP1-145E was identified as a major determinant of viremia and neuropathogenesis, and may play a key role in the *in vivo* fitness of EV71. On the other hand, VP1-145G variants were identified only in PBMCs as viral quasi-species, indicating the possible involvement of PSGL-1-dependent EV71 replication in the local cell microenvironment. In this regard, our study provides new insights into the interplay between virus (tissue- and cell-tropic EV71 variants), receptors (PSGL-1, SCARB2, and other EV71 binding molecules), and host (immune responses and pathogenesis) in EV71-infected individuals.

## Materials and Methods

### Ethics statement

Animal experiments were conducted in compliance with Japanese legislation (Act on Welfare and Management of Animals, 1973, revised in 2012) and guidelines under the jurisdiction of the Ministry of Education, Culture, Sports, Science and Technology, Japan (Fundamental Guidelines for Proper Conduct of Animal Experiment and Related Activities in Academic Research Institutions, 2006). Animal care, housing, feeding, sampling, observation, and environmental enrichment were performed in accordance with the guidelines. Every possible effort was made to minimize animal suffering. The protocols of animal experiments were approved by the committee of biosafety and animal handling and the committee of ethical regulation of the National Institute of Infectious Diseases, Japan (Authorization Number; 512001). Each animal was housed in a separated cage, received standard primate feed and fresh fruit daily, and had free access to water at the National Institute of Infectious Diseases, Japan, and animal welfare was observed on a daily basis. Sampling procedures were conducted under anesthesia (10 mg/kg ketamine intramuscularly). Animals were sacrificed under excess anesthesia.

### Cells

Jurkat T cells were obtained from the Riken Cell Bank and cultured in RPMI-1640 medium (Sigma, St. Louis, MO) supplemented with 10% fetal bovine serum (FBS; CCB Nichirei Bioscience, Japan). RD cells were obtained from the US Centers for Disease Control and maintained in MEM (Sigma, St. Louis, MO) supplemented with 10% FBS.

### Infectious viral cDNA clones

We used the genomic RNA of the 02363 strain of EV71 (non-PB; subgenogroup C1) as the template for RT-PCR. Infectious viral cDNA clones were constructed and cDNA-derived mutant viruses were prepared as previously described [30]. The RNA-transfected cells and supernatants were freeze-thawed thrice at 24 h post-transfection. Before use in experiments, recovered viruses were amplified once in fresh RD cells, and the sequence of the whole capsid region was confirmed by direct sequencing of RT-PCR products. Sequence identity between the virus stock and original cDNA was confirmed. Viral titers were determined by a microtitration assay using 96-well plates and RD cells, as previously described [62]. Briefly, 10 wells were used for each viral dilution and viral titers were expressed as 50% cell culture infectious dose (CCID_50_).

### Animals

Eight male cynomolgus macaques (*Macaca fascicularis*) imported from the Philippines were used in this study. Seven out of eight monkeys were 4 years old and the other was 6 years old (#5061). The average weight was 3.0 kg (range, 2.4 to 4.3 kg). All procedures were performed in a biosafety level 2 containment facility. Serological testing revealed all animals used in this study were free from infection with EV71 and poliovirus.

### Inoculations

Eight monkeys were divided into two groups; one group received EV71-02363-EG (PB) inoculations (monkeys No. #5131, #5134, #5135, and #5136) and the other group received EV71-02363-KE (non-PB) inoculations (monkey No. #5061, #5132, #5133, and #5137). Virus inoculation and sampling procedures were conducted under anesthesia (10 mg/kg ketamine intramuscularly). One ml of EV71 virus solution containing either 10^6.3^ CCID_50_ of 02363-KE (non-PB) or 02363-EG (PB) strain was intravenously inoculated into the right tibial vein. Following inoculation, monkeys were observed daily for 10 days to assess neurological manifestations. Clinical manifestations were observed in more detail under anesthesia, including blisters within the oral cavity, palm, and sole of the foot, at days 0, 3, 7 and 10 p.i. Whole blood, throat, and rectal swab samples were collected at these times. CSF samples were collected at 7 and 10 days p.i. Blood-contaminated CSF samples were excluded from the analysis (S2 Table). Isolation of PBMC and serum from whole blood was carried out within one day of collection.

Monkeys were sacrificed under deep anesthesia at 10 days p.i. and various parts of the CNS, main organs, lymph nodes, swabs and blood samples were collected for histopathological and virological analyses. Ten percent [wt/vol] homogenates of collected tissues were prepared in Eagle’s minimal essential medium (MEM) containing 2% FBS with MagNA Lyzer (Roche, Basel, CH) before centrifugation at 10,000 × *g* for 5 min at 4°C to remove tissue debris. The supernatants were used for virus isolation in RD cells and RNA extraction for molecular detection of EV71.

### Sequence analysis of the VP1 region of EV71

To investigate the genetic stability of EV71 strains following inoculation, we determined the entire and/or partial VP1 sequence of EV71 from clinical and postmortem samples. To minimize selection and adaptation during cell culture, the entire VP1 region of EV71 was directly amplified from the samples by RT-PCR without virus isolation. Briefly, before RNA extraction, swab samples were centrifuged at 2000 rpm for 3 min and the supernatants were used for RNA extraction. Viral genomic RNA was extracted from 10% tissue homogenates of autopsy or clinical samples using HighPure viral RNA purification kit (Roche, Penzberg, DE). Reverse transcription-PCR (RT-PCR) was performed using PrimeScript II High Fidelity RT-PCR Kits (Takara, Japan). PCR products were purified using Wizard SV Gel and PCR Clean-Up System PCR purification kits (Promega, Madison, USA). The full length of the VP1 sequence was analyzed using the following primers;

VP1-1: 5′-TAATAGCACTAGCGGCAGCC-3′,

VP1-2: 5′-AGCTGAGACCACTCTCGATAG-3′,

VP1-3R: 5′-TGGGGTATCCGTCATAGAACC-3′

VP1-4R: 5′-TGGTGGATGACACGAGCAAG-3′

The full length of the VP1 sequence was amplified as a series of overlapping fragments using primer sets VP1-1/VP1-3R (714 nt) and VP1-2/VP1-4R (803 nt). All primers were used in sequencing analysis. When the entire VP1 fragment was not amplified by RT-PCR directly from samples, a highly sensitive CODEHOP RT-PCR was used to amplify the partial VP1 region [49]. Briefly, cDNA was synthesized from total RNA using four primers (AN32–AN35). The partial VP1 region was amplified by semi-nested PCR: primers 222 and 224 were used for the first-round PCR and primers AN88 and AN89 for the second amplification. DNA sequencing was performed using BigDye Terminator v3.0 cycle sequencing ready reaction kits (Applied Biosystems, Foster city, CA) and results were analyzed using an ABI 3130 genetic analyzer (Applied Biosystems, Foster City, CA) and Sequencher ver. 5.0.1 sequence analysis software (Gene Codes Corporation, Ann Arbor, MI).

### Quasi-species analysis

To assess quasi-species at VP1-98 and VP-145, partial capsid cDNA (about 700 nt covering VP3-214 to VP1-210) was amplified by VP1-1 and VP1-3R primers directly from tissue or clinical samples and cloned into a pCR-TOPO vector using TOPO-TA cloning kit (Life technologies, Carlsbad, CA) according to the manufacturer’s instructions. Resultant colonies were randomly selected and plasmids were purified using QIAprep Miniprep kits (QIAGEN, Hilden, Germany). In total, forty-four independent plasmids carrying viral cDNA from 7 postmortem samples (3 CNS, 3 non-CNS, and 1 PBMC samples) from 02363-KE- or 02363-EG-inoculated monkeys were sequenced. Similarly, thirty independent plasmids derived from 4 clinical samples (2 rectal and 2 throat swab samples) from two 02363-KE-inoculated monkeys were analyzed. Viral quasi-species variants were identified by nucleotide sequence alignment of plasmid clones derived from each clinical or tissue sample.

### Virus isolation

For virus isolation, RD cells inoculated with tissue homogenates or clinical sample preparations were observed for cytopathic effect (CPE) for one week and then blind passage was performed for CPE-negative samples after freeze-thawing of the first-round passage. If no CPE were observed during first and second round cultures, the result of virus isolation was recorded as negative.

### Neutralizing antibody titers in monkey serum

Serum samples were diluted serially two-fold (1:4–1:4096) in MEM containing 2% FBS. The EV71 stock was diluted to a concentration of 100 CCID_50_/50 μl. The 50 μl of diluted serum was mixed with 50 μl of EV71 solution on 96-well plates in duplicate and incubated for 2 h at 35°C. Following incubation, 100 μl of the cell suspension containing 10^4^ RD cells was added to the wells followed by incubation at 35°C with 5% CO_2_ for 7 days to observe CPE. Neutralization titers were determined as the highest serum dilutions that could completely protect from CPE. Neutralization titers against the 02363-KE strain were difficult to determine, presumably due to non-specific viral aggregation. Therefore, to minimize aggregation, virus solutions were pre-treated with an equal volume of chloroform, shook vigorously for 20 min, and then centrifuged at 10,000 × *g* for 10 min at 4°C. Supernatants were used as the challenge virus for the neutralization assay. Cross neutralization titers were measured using serum samples collected at 10 days p.i.

### Isolation of PBMC from healthy monkeys

PBMCs were isolated from 5 ml of whole blood from cynomolgus monkeys mixed with EDTA for anti-coagulation by Percoll density-gradient centrifugation. Briefly, whole blood was diluted with PBS and carefully layered over 5 ml of Percoll (specific gravity 1.070 g/ml) in 15 ml conical tubes. Tubes were centrifuged at 400 × *g* for 30 min at 20°C in a swinging-bucket rotor. Mononuclear cell layers were carefully transferred to a new tube, mixed with PBS, and centrifuged at 300 × *g* for 10 min at 20°C before complete removal of supernatants. To remove platelets, cells were resuspended in PBS and centrifuged at 200 × *g* for 10 min at 20°C before complete removal of supernatants. This procedure was repeated once. Precipitated cells were used for RNA extraction, infection assays, and CD14-positive cell isolation.

### Isolation of CD14-positive cells

CD14-positive cells were isolated from freshly isolated PBMC using CD14 microbeads and MACS MS columns according to the manufacturer’s instructions (Miltenyi Biotec, Bergisch Gladbach, Germany). Isolated CD14-positive cells were cultured in RPMI-1640 with 10% FBS.

### EV71 infection of PBMC and CD14-positive cells from healthy monkeys

Monkey PBMC and CD14-positive cells were inoculated with 02363-KE or 02363-EG strain at 1 CCID_50_/cell and incubated at 35°C with 5% CO_2_ for 2 h. After incubation, cells were washed with RPMI-1640 without FBS twice and then resuspended with RPMI-1640 containing 10% FBS. Cells were cultured in 96-well plates at 35°C with 5% CO_2_. CD14-positive cells were harvested at 0, 1 and 3 days p.i. PBMCs were harvested at 0, 1, 3 and 7 days after infection. Cells and supernatants were collected together for RNA extraction. To monitor EV71 viral replication in PBMC and CD14-positive cells, viral genomic RNA in each cell preparation was measured by real-time PCR using the following primers: EnteroFw, 5′-GCCCCTGAATGCGGCTAAT-3′ and EnteroRev, 5′-ATTGTCACCATAAGCAGCCA-3′, as described previously [63].

### Histopathology and immunohistochemistry

Brain, spinal cord, lung, heart, liver, spleen, kidney, lymph nodes, tonsil, and gastrointestinal tract samples were fixed in 10% formalin in PBS and embedded in paraffin. Paraffin-embedded sections were stained with hematoxylin and eosin (H&E). Immunohistochemical detection of the EV71 antigen was performed on paraffin-embedded sections using a labeled streptavidin-biotin method (Dako Denmark A/S, Glostrup, Denmark). For antigen retrieval, sections were heated to 121°C for 10 min in an autoclave with 10 mM citrate buffer solution (pH 6.0). A polyclonal antibody raised against denatured virus particles from the 1095/Japan 97 isolate of EV71 was used as the primary antibody [44]. Peroxidase activity was detected with 3, 3-diaminobenzidine (Sigma-Aldrich, St. Louis, MO) and sections were counterstained with hematoxylin.

### Detection of lymphocytes in CSF

Lymphocytes in CSF were detected by VetScan HM2 (ABAXIS, Union City, CA) according to manufacturer’s instructions. One hundred μl of each CSF sample was used for Giemsa staining as following: cells were attached to glass slides using a Shandon cytocentrifuge (Thermo Fisher Scientific Inc., Waltham, MA) at 1,000 rpm for 10 min and then stained with Giemsa’s Azure Eosin Methylene Blue solution (Merck Millipore, Billerica, MA). Stained cells were analyzed by microscopy.

### Flow cytometric analyses

The number of lymphocytes in peripheral blood samples from infected monkeys was calculated by flow cytometry. Both CD3^+^CD4^+^ and CD3^+^CD8^+^ T cells were stained using NHP T Lymphocyte Cocktail (BD Pharmingen, San Diego, CA) and CD3^-^CD16^+^ NK cells and CD3^-^CD20^+^ B cells were stained using NHP T/B/NK Cell Cocktail (BD Pharmingen, San Diego, CA). Analyses were performed using a BD FACSCanto II Flow Cytometer.

### Cytokine and chemokine analyses

Serum cytokine concentrations were analyzed using Cytokine Monkey Magnetic 28-Plex Panels (Life technologies, Carlsbad, CA) on a Luminex 200 (Luminex, Austin, TX).

### Statistical analyses

We carried out flow cytometric (Fig 3 and S3 Fig) and cytokine/chemokine (Fig 4 and S4 Fig) assays in triplicate and compared mean values using Student’s *t*-test. *P* < 0.05 were considered statistically significant.

## Supporting Information

S1 Fig Serum neutralization titers against EV71 in inoculated monkeys. The neutralizing activity against the inoculated homotypic EV71 strain was determined in serum samples from each monkey collected on preinfection (Day 0), and 3, 7, and 10 days postinfection by microneutralization assay.
(PDF)

S2 Fig Comparative analysis of peripheral blood lymphocytes from monkeys inoculated with the 02363-KE (non-PB) and 02363-EG (PB) strains. Average numbers of (A) CD3^+^CD4^+^ T-lymphocytes, (B) CD3^+^CD8^+^ T-lymphocytes, (C) CD3^-^CD16^+^ lymphocytes (NK cells), and (D) CD3^-^CD20^+^ B-lymphocytes in peripheral blood collected from monkeys inoculated with EV71-02363-KE (non-PB; shaded in blue) or EV71-02363-EG (PB; shaded in orange) strain are shown. Significant differences in lymphocyte numbers between 02363-KE- and 02363-EG-inoculated groups at indicated days were determined using the same data set in Fig 3. No significant differences (*P*<0.05) were observed between the two groups.
(PDF)

S3 Fig Comparative analysis of serum cytokine levels in monkeys inoculated with the 02363-KE (non-PB) and 02363-EG (PB) strains. Average of serum cytokine concentrations of, (A) IL-1β, (B) TNF-α, (C) IL-6, (D) G-CSF, (E) IL-1RA, and (F) IFN-γ collected from monkeys inoculated with EV71-02363-KE (non-PB; shaded in blue) or EV71-02363-EG (PB; shaded in orange) strain are shown. ND (not detected) indicates that serum cytokine levels of all samples are below the limit of detection. Significant differences in cytokine levels between 02363-KE- and 02363-EG-inoculated groups at indicated days were determined using the same data set in Fig 4. Significant differences (*P* < 0.05) between the two groups are indicated by asterisks.
(PDF)

S4 Fig Giemsa staining of lymphocytes in CSF. CSF samples collected at 7 days postinfection from (A) monkey #5132, inoculated with 02363-KE (non-PB), and (B) monkey #5135, inoculated with 02363-EG (PB). Typical meningitis (lymphocytosis in CSF) was observed in monkey #5132 but not in monkey #5135 (see also S2 Table).
(PDF)

S5 Fig Replication kinetics of 02363-KE (non-PB) and 02363-EG (PB) strains in PBMC or CD14-posortive cells from healthy monkeys. Monkey PBMC (A) and CD14-positive cells (B) were inoculated with 02363-KE or 02363-EG strain at 1 CCID_50_/cell and viral genomic RNA in each cell preparation was measured by real-time PCR to monitor EV71 viral replication. ND; viral RNA could not be detected. Means of RNA copy numbers ± SD in four PBMC preparations (A) and in three CD14-positive cell preparations (B) are indicated.
(PDF)

S6 Fig Cross-neutralization titers in inoculated monkeys. Serum samples were collected at 10 days postinfection from 02363-KE- (non-PB: shaded in blue) and 02363-EG- (PB: shaded in orange) inoculated monkeys. Serum neutralization titers against the 02363-KE (upper) and 02363-EG (lower) strains were measured to assess homotypic and cross-neutralization titers.
(PDF)

S1 Table Quasi-species at VP1-98 and VP1-145. To assess quasi-species at VP1-98 and VP-145, partial capsid cDNA was amplified directly from tissue or clinical samples and cloned into plasmid vectors. Each plasmid was sequenced and sequences were aligned to identify quasi-species in the same sample. Nucleotide and amino acid substitutions from the original inoculated viruses are indicated in red.
(PDF)

S2 Table Number of lymphocytes in CSF samples. Blood-contaminated CSF samples (ND) were excluded from the analysis. Lymphocyte numbers were measured by using the VetScan HM2.
(PDF)
