# Supplementary figures and images for: The Role of VP1 Amino Acid Residue 145 of Enterovirus 71 in Viral Fitness and Pathogenesis in a Cynomolgus Monkey Model
Source: PLoS Pathog. 2015 Jul 16;11(7):e1005033. doi: 10.1371/journal.ppat.1005033 (PMC4504482; doi:10.1371/journal.ppat.1005033)

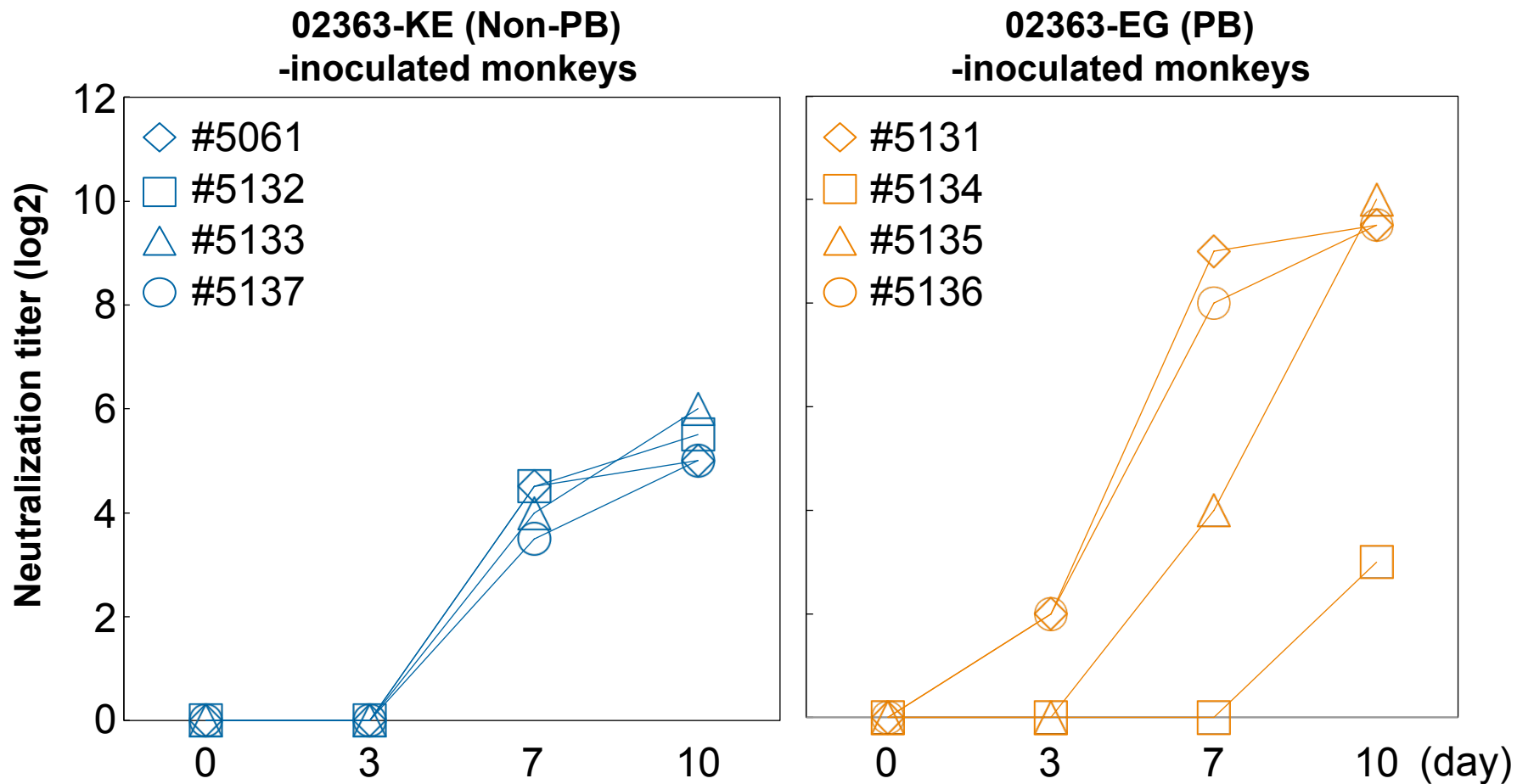

Supplement: S1 Fig — The neutralizing activity against the inoculated homotypic EV71 strain was determined in serum samples from each monkey collected on preinfection (Day 0), and 3, 7, and 10 days postinfection by microneutralization assay. (PDF) [file ppat.1005033.s001.pdf]

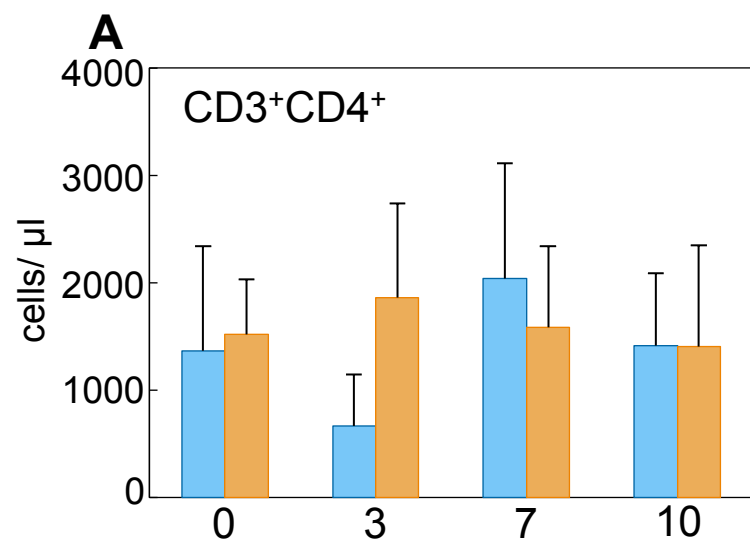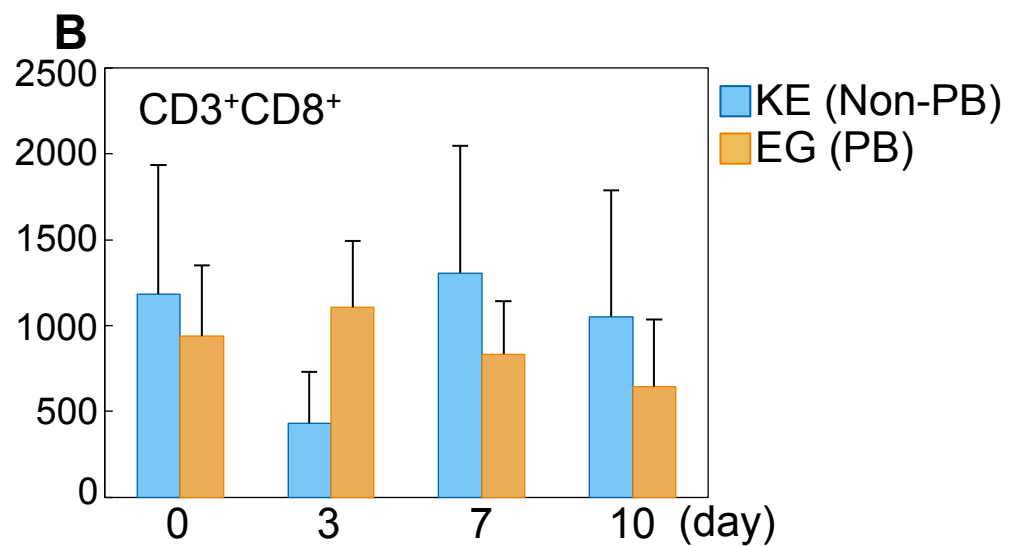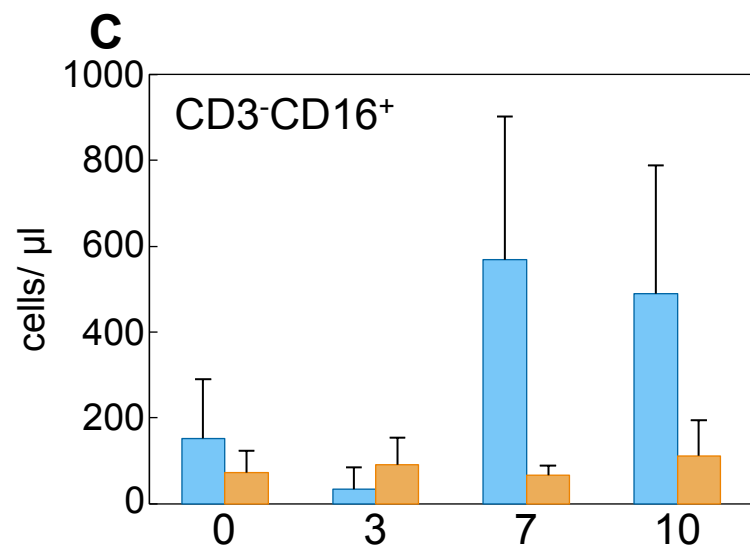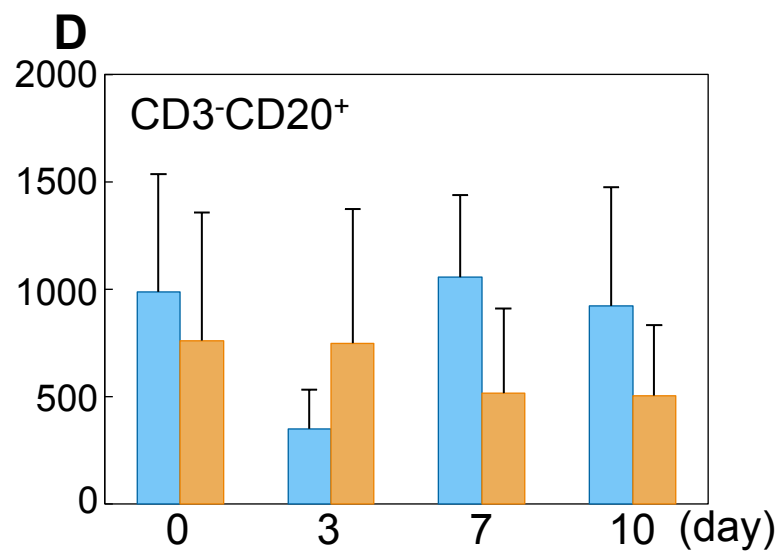

Supplement: S2 Fig — Average numbers of (A) CD3+CD4+ T-lymphocytes, (B) CD3+CD8+ T-lymphocytes, (C) CD3-CD16+ lymphocytes (NK cells), and (D) CD3-CD20+ B-lymphocytes in peripheral blood collected from monkeys inoculated with EV71-02363-KE (non-PB; shaded in blue) or EV71-02363-EG (PB; shaded in orange) strain are shown. Significant differences in lymphocyte numbers between 02363-KE- and 02363-EG-inoculated groups at indicated days were determined using the same data set in Fig 3. No significant differences (P<0.05) were observed between the two groups. (PDF) [file ppat.1005033.s002.pdf]

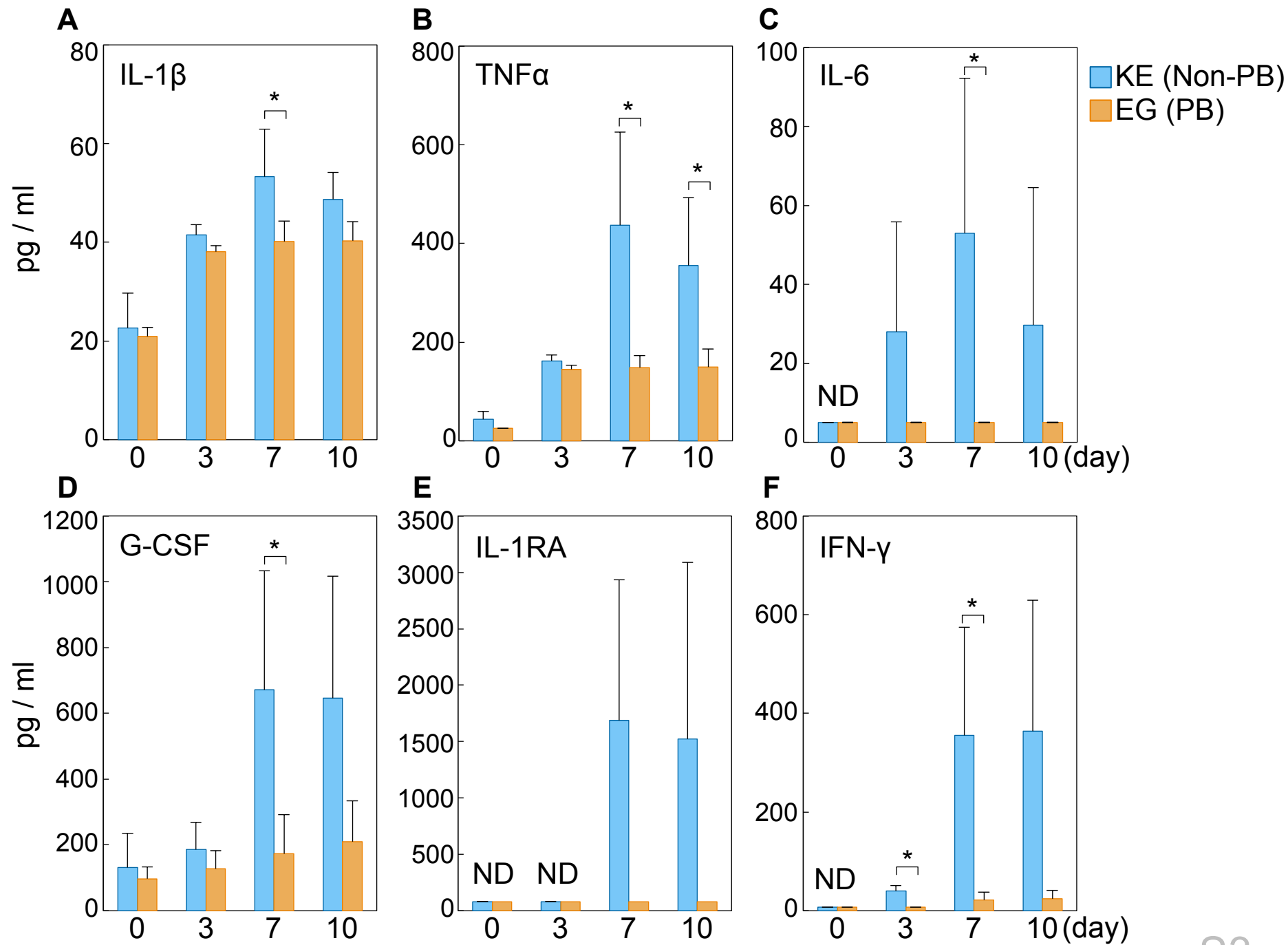

Supplement: S3 Fig — Average of serum cytokine concentrations of, (A) IL-1β, (B) TNF-α, (C) IL-6, (D) G-CSF, (E) IL-1RA, and (F) IFN-γ collected from monkeys inoculated with EV71-02363-KE (non-PB; shaded in blue) or EV71-02363-EG (PB; shaded in orange) strain are shown. ND (not detected) indicates that serum cytokine levels of all samples are below the limit of detection. Significant differences in cytokine levels between 02363-KE- and 02363-EG-inoculated groups at indicated days were determined using the same data set in Fig 4. Significant differences (P < 0.05) between the two groups are indicated by asterisks. (PDF) [file ppat.1005033.s003.pdf]

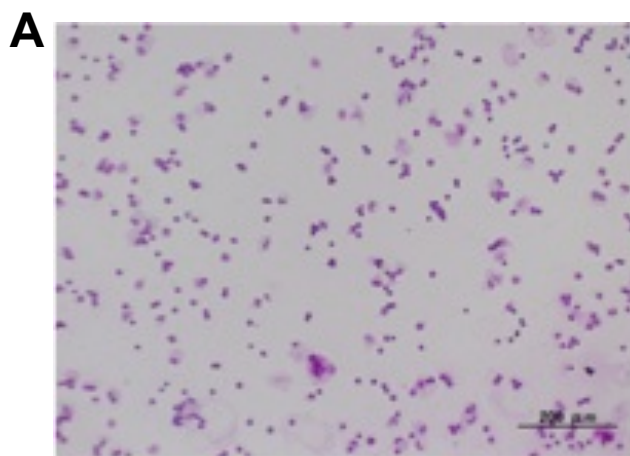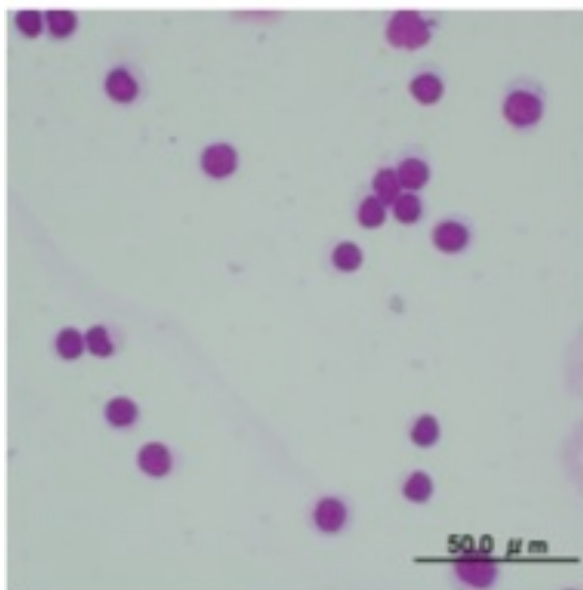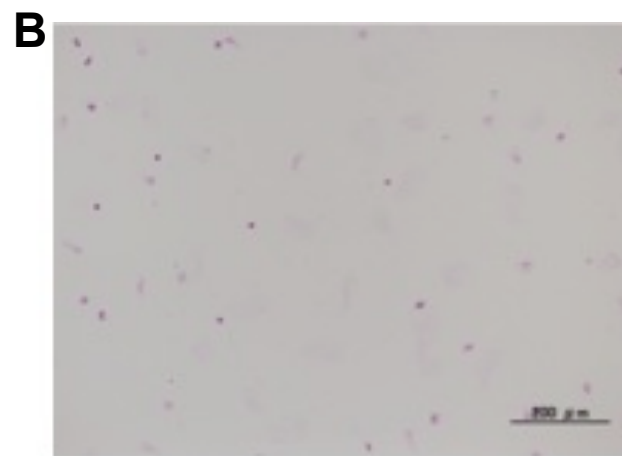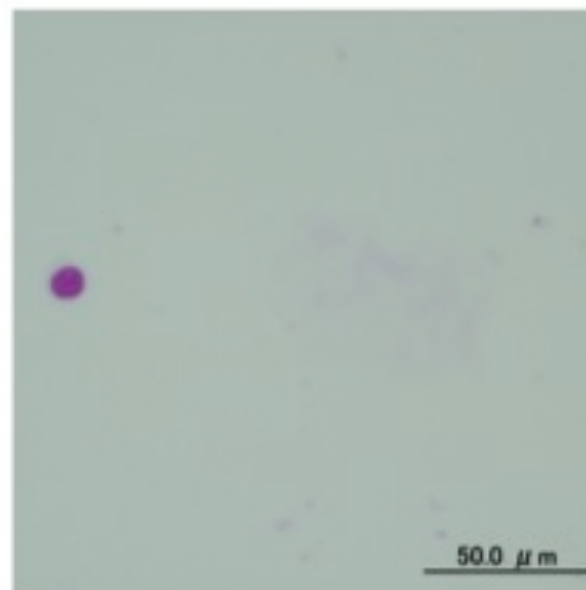

Supplement: S4 Fig — CSF samples collected at 7 days postinfection from (A) monkey #5132, inoculated with 02363-KE (non-PB), and (B) monkey #5135, inoculated with 02363-EG (PB). Typical meningitis (lymphocytosis in CSF) was observed in monkey #5132 but not in monkey #5135 (see also S2 Table). (PDF) [file ppat.1005033.s004.pdf]

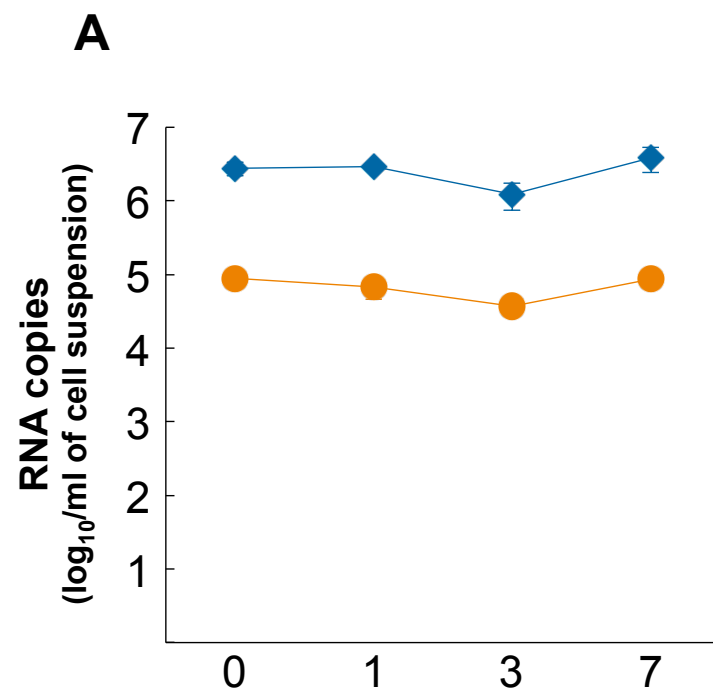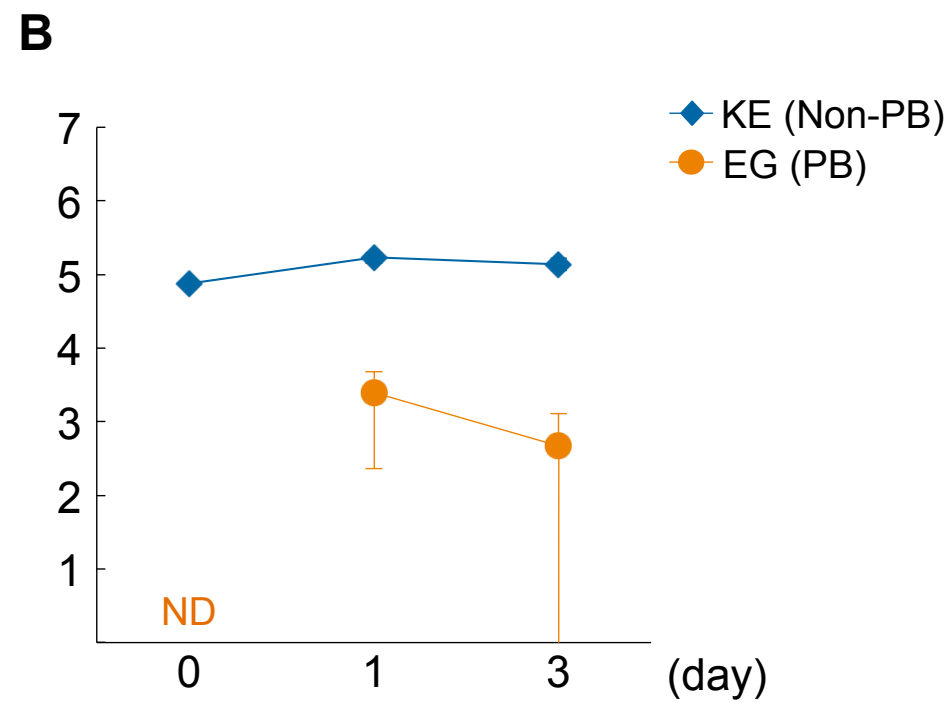

Supplement: S5 Fig — Monkey PBMC (A) and CD14-positive cells (B) were inoculated with 02363-KE or 02363-EG strain at 1 CCID50/cell and viral genomic RNA in each cell preparation was measured by real-time PCR to monitor EV71 viral replication. ND; viral RNA could not be detected. Means of RNA copy numbers ± SD in four PBMC preparations (A) and in three CD14-positive cell preparations (B) are indicated. (PDF) [file ppat.1005033.s005.pdf]

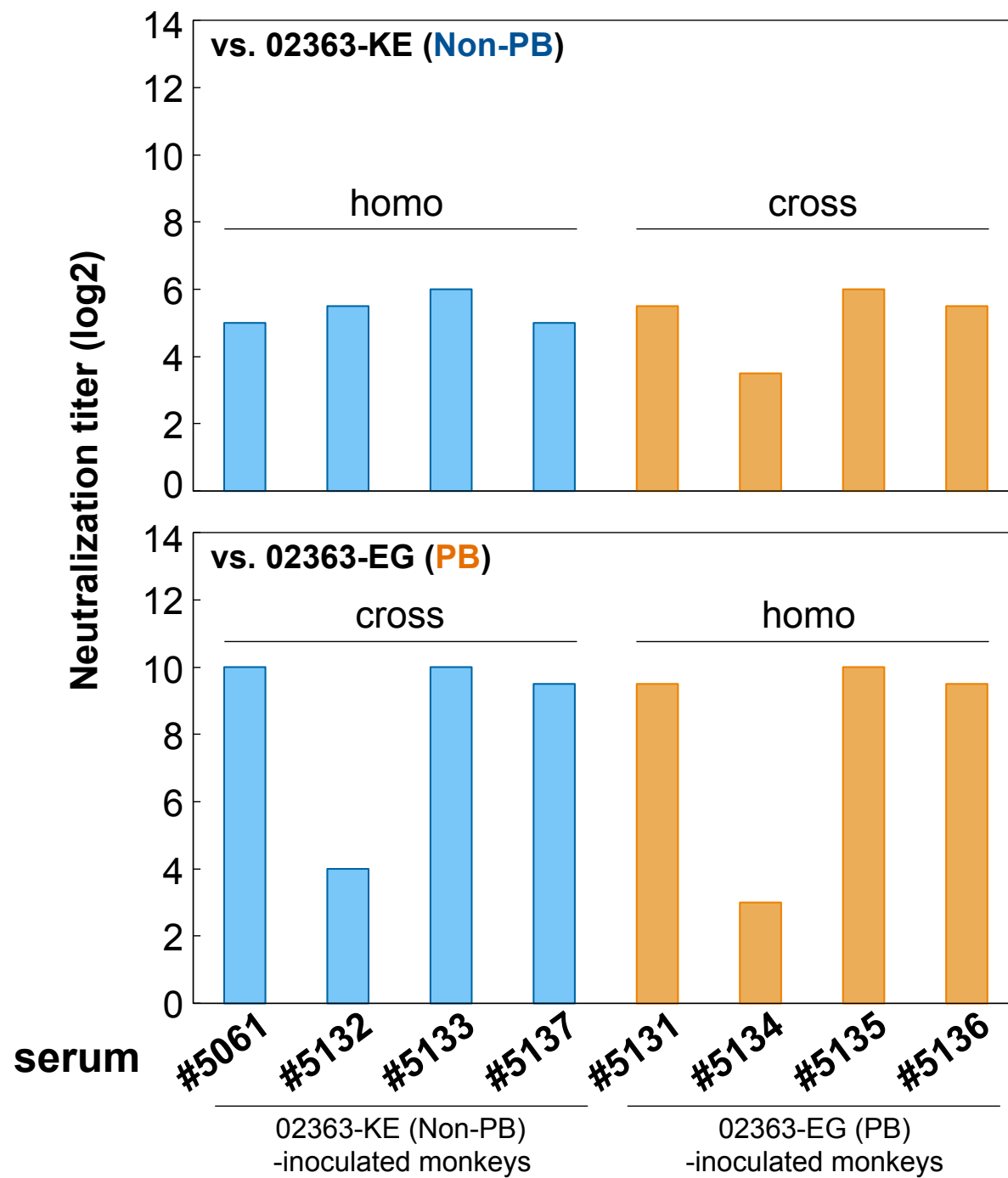

S6 Figure

Supplement: S6 Fig — Serum samples were collected at 10 days postinfection from 02363-KE- (non-PB: shaded in blue) and 02363-EG- (PB: shaded in orange) inoculated monkeys. Serum neutralization titers against the 02363-KE (upper) and 02363-EG (lower) strains were measured to assess homotypic and cross-neutralization titers. (PDF) [file ppat.1005033.s006.pdf]
