# Supplementary material for: The Role of VP1 Amino Acid Residue 145 of Enterovirus 71 in Viral Fitness and Pathogenesis in a Cynomolgus Monkey Model
Source: PLoS Pathog. 2015 Jul 16;11(7):e1005033. doi: 10.1371/journal.ppat.1005033 (PMC4504482; doi:10.1371/journal.ppat.1005033)
Supplement: S1 Table — To assess quasi-species at VP1-98 and VP-145, partial capsid cDNA was amplified directly from tissue or clinical samples and cloned into plasmid vectors. Each plasmid was sequenced and sequences were aligned to identify quasi-species in the same sample. Nucleotide and amino acid substitutions from the original inoculated viruses are indicated in red. (PDF) [file ppat.1005033.s007.pdf]

# S1 Table Quasi-species at VP1-98 and VP1-145

| Monkey No. | Inoculated virus | Clinical or tissues samples | | Summary of sequence analysis | | | | | Remarks |
|---|---|---|---|---|---|---|---|---|---|
| | | Samples | Date of sampling | Direct sequence | | Sequence of the cloned plasmids | | | |
| | | | | VP1-98 | VP1-145 | No. of clones sequenced | VP1-98 | VP1-145 | |
| #5137 | 02363-KE (non-PB) | Rectal swab | 3 days pi | K (AAA) | E (GAG) | 7 | K (AAA): 2<br>**Q** (**C**AA): 2<br>**N** (AA**T**): 2<br>**E** (**G**AA): 1 | E (GAG): 7 | Quasi-species was identified at VP1-98 (K/**Q**/**N**/**E**) |
| #5137 | 02363-KE (non-PB) | Rectal swab | 10 days pi | **E** (**G**AA) | E (GAG) | 8 | **E** (**G**AA): 7<br>**N** (AA**T**): 1 | E (GAG): 8 | Quasi-species was identified at VP1-98 (**E**/**N**) |
| #5137 | 02363-KE (non-PB) | Throat swab | 3 days pi | K (AAA) | E (GAG) | 7 | K (AAA): 4<br>**Q** (**C**AA): 2<br>**N** (AA**T**): 1 | E (GAG): 7 | Quasi-species was identified at VP1-98 (K/**Q**/**N**) |
| #5133 | 02363-KE (non-PB) | Throat swab | 3 days pi | **E** (**G**AA) | E (GAG) | 8 | **E** (**G**AA): 7<br>**N** (AA**T**): 1 | E (GAG): 8 | Quasi-species was identified at VP1-98 (**E**/**N**) |
| #5132 | 02363-KE (non-PB) | Cervical code | 10 days pi | **E** (**G**AA) | E (GAG) | 9 | **E** (**G**AA): 9 | E (GAG): 9 | All the 9 plasmid clones contained a substitution from VP1-98K (AAA) to VP1-98**E** (**G**AA) |
| #5136 | 02363-EG (PB) | Cervical code | 10 days pi | E (GAA) | **E** (G**A**G) | 8 | E (GAA): 8 | **E** (G**A**G): 8 | All the 8 plasmid clones contained a substitution from VP1-145G (GGG) to VP1-145**E** (G**A**G) |
| #5136 | 02363-EG (PB) | Medulla oblongata | 10 days pi | E (GAA) | **E** (G**A**G) | 5 | E (GAA): 5 | **E** (G**A**G): 5 | All the 5 plasmid clones contained a substitution from VP1-145G (GGG) to VP1-145**E** (G**A**G) |
| #5134 | 02363-EG (PB) | Spleen | 10 days pi | E (GAA) | **E** (G**A**G) | 8 | E (GAA): 8 | **E** (G**A**G): 8 | All the 5 plasmid clones contained a substitution from VP1-145G (GGG) to VP1-145**E** (G**A**G) |
| #5133 | 02363-KE (non-PB) | Deep cervical lymph node | 10 days pi | **E** (**G**AA) | E (GAG) | 7 | **E** (**G**AA): 7 | E (GAG): 7 | All the 7 plasmid clones contained a substitution from VP1-98K (AAA) to VP1-98**E** (**G**AA) |
| #5134 | 02363-EG (PB) | Deep cervical lymph node | 10 days pi | E (GAA) | **E** (G**A**G) | 7 | E (GAA): 7 | **E** (G**A**G): 7 | All the 7 plasmid clones contained a substitution from VP1-145G (GGG) to VP1-145**E** (G**A**G) |
| #5136 | 02363-EG (PB) | PBMC | 10 days pi | E (GAA) | G (GGG) | 9 | E (GAA): 9 | G (GGG): 8<br>**E** (G**A**G): 1 | Quasi-species was identified at VP1-145 (G/**E**) |

To assess quasi-species at VP1-98 and VP-145, partial capsid cDNA was amplified directly from tissue or clinical samples and cloned into a plasmid vector. Each plasmid was sequenced and the sequences were aligned to identify quasi-species in the same sample. Nucleotide and amino acid substitutions from the original inoculated viruses are indicated in bold.
