# Supplementary material for: The Role of VP1 Amino Acid Residue 145 of Enterovirus 71 in Viral Fitness and Pathogenesis in a Cynomolgus Monkey Model
Source: PLoS Pathog. 2015 Jul 16;11(7):e1005033. doi: 10.1371/journal.ppat.1005033 (PMC4504482; doi:10.1371/journal.ppat.1005033)
Supplement: S2 Table — Blood-contaminated CSF samples (ND) were excluded from the analysis. Lymphocyte numbers were measured by using the VetScan HM2. (PDF) [file ppat.1005033.s008.pdf]

S2 Table Number of lymphocytes in CSF samples

CSF sample	Concentration of lymphocytes (cells/µl CSF)							
	02363-KE (Non-PB)-inoculated monkey				02363-EG (PB)-inoculated monkey			
Days post-infection	#5061	#5132	#5133	#5137	#5131	#5134	#5135	#5136
7	ND	130	ND	170	ND	0	0	ND
10	250	20	160	280	0	ND	ND	ND

Blood-contaminated CSF samples (ND) were excluded from the analysis. Lymphocyte numbers were measured by using the VetScan HM2.
